# Effects of Sodium Glucose Co-Transporter 2 Inhibitors on Atrial Fibrillation Recurrences After Catheter Ablation in Atrial Fibrillation Patients: A Systematic Review and Meta-Analysis

**DOI:** 10.3390/jcm14228001

**Published:** 2025-11-11

**Authors:** Saketh Parsi, Kunal Sonavane, Usha Ravi, Pallavi D. Shirsat, Venkata S. Chamarthi, Mohamed Gabr, Harikrishna Choudary Ponnam, Salim Surani, Vikas Bansal, Rahul Kashyap

**Affiliations:** 1 Department of Internal Medicine, Ascension Seton Medical Center Austin, Austin, TX 78705, USA; 2 Department of Internal Medicine, Willis Knighton Health, Bossier City, LA 71111, USA; 3 Department of Pediatrics, Pediatric Associates, Visalia, CA 93277, USA; drusharavi25@gmail.com; 4 Department of Nephrology, Minden Medical Center, Minden, LA 71055, USA; 5 Department of Pediatrics, Valley Children’s Healthcare, Fresno, CA 93720, USA; vchamarthi1@valleychildrens.org; 6 Cardiac Arrhythmia Service, Brigham and Women’s Hospital, Harvard Medical School, Boston, MA 02115, USA; mgabr@bwh.harvard.edu; 7 Department of Internal Medicine, Summa Health System, Akron, OH 44304, USA; drponnam@gmail.com; 8 Department of Medicine & Pharmacology, Texas A & M, College Station, TX 77840, USA; srsurani@gmail.com; 9 Department of Research, WellSpan Health, York, PA 17403, USA; drvikasbansal@gmail.com (V.B.); kashyapmd@gmail.com (R.K.); 10 Department of Anesthesia and Critical Care Medicine, Mayo Clinic, Rochester, MN 55905, USA

**Keywords:** sodium glucose co-transporter 2 inhibitor, atrial fibrillation, ablation, atrial fibrillation recurrence, cardiac remodeling

## Abstract

**Background/Objectives:** Sodium glucose co-transporter 2 inhibitors (SGLT2is) have demonstrated a reduction in heart failure (HF) hospitalizations in HF patients and decreased recurrence of atrial fibrillation (AF), including in those who have undergone catheter ablation (CA). The effects of SGLT2i are likely due to suppression of the renin–angiotensin–aldosterone system, reduction in oxidative stress with subsequent improvement in myocardial efficiency, and attenuation of cardiac remodeling. We aim to present the effects of SGLT2i on AF recurrence in patients who have undergone CA for AF. **Methods:** This is a systematic review and meta-analysis of randomized controlled trials (RCTs) and retrospective studies evaluating the effect of SGLT2i on AF recurrence following CA compared with non-SGLT2i. The primary outcome was the recurrence of AF by the final follow-up reported in each study. Secondary outcomes include AF recurrence by the first follow-up within 12 to 24 months and follow-up intervals (6, 12, 18, 24, and 36 to 42 months) post-ablation, multivariate risk of AF recurrence, and the effect on left atrial diameter (LAD) (less than 45 mm vs. greater than or equal to 45 mm). For risk of bias (ROB) analysis, the NIH ROB and Cochrane ROB2 tool were used. All statistical, heterogeneity, and sensitivity analyses were conducted using Cochrane Review Manager. A random-effect model was employed for all pooled statistical analyses. **Results:** A total of nine studies, two RCTs and seven retrospective studies, were included (N = 6874) for the primary outcome. Compared to non-SGLT2i (N = 3693), SGLT2i (N = 3181) significantly decreased AF recurrence by the final follow-up (OR = 0.62; 95% CI: 0.45–0.85; *p* = 0.008). For secondary outcomes, SGLT2i significantly reduced AF recurrence by the first follow-up within 12 to 24 months post-ablation (OR = 0.58; *p* = 0.0001) and by the different follow-up periods, 6-month (OR = 0.53; *p* = 0.02), 12-month (OR = 0.56; *p* = 0.0001), 18-month (OR = 0.55; *p* = 0.01), and 24-month (OR = 0.60; *p* = 0.12) follow-up periods. On the other hand, by 36 to 42 months, SGLT2i was associated with increased risk of AF recurrence (OR = 1.41; *p* = 0.004). **Conclusions:** We conclude that SGLT2i demonstrated a reduction in AF recurrence following CA, particularly by 12 to 18 months post-ablation.

## 1. Introduction

Atrial fibrillation (AF) is the most common cardiac arrhythmia, affecting 33.5 million people globally [1]. It affects between 2.7 to 6.1 million people in the United States (US), with the prevalence estimated to rise to 12.1 million by 2030 [2]. AF is associated with significantly increased risks of hospitalization, heart failure (HF), and cardiovascular events, translating into a significant financial burden to the patients and the healthcare system. The cumulative AF-related healthcare expenditure in the US is around $22.1 billion per year, and is expected to rise to $65 billion by 2035 [3]. Risk factors for AF, amongst others, include heart failure, valvular heart disease, hypertension, diabetes mellitus (DM), thyroid dysfunction, inflammatory conditions such as myocarditis or pericarditis, smoking or alcohol use, advancing age, obesity, obstructive sleep apnea, and genetic predisposition [4].

Management strategies for AF have evolved over the years. Broadly, management of AF falls into the following categories: rate control, rhythm control, prevention, and management of comorbidities. Catheter ablation (CA) has become an established treatment modality for symptomatic AF. AF ablation procedure volume in the US has increased from 83.8 in 2017 to 111.6 per 100,000 patient-years in 2021 and is on the rise [5]. Nonetheless, rates of freedom from AF recurrence after CA remain suboptimal. A prior study looking at long-term outcomes of CA in the AF population showed a 1-year success rate of 65.3%, which decreased to 56.4% at 3 years and incrementally dropped to 51.2% at 5-year follow-up [6], and recurrence-free survival probability at 5-year follow-up of 59.4% in another study after initial CA [7]. Contemporary studies similarly reveal 12-month recurrence rates of around 10–20% in paroxysmal AF and 45–50% for persistent AF [8,9]. Based on a recent study of 12,000 patients who underwent CA for AF [10], repeat ablation carried out within 1 year in 17% of patients was associated with higher medical cost compared to patients who did not undergo repeat ablation ($52,821 vs. $13,412). Patients who underwent repeat ablation had higher rates of emergency department visits (43.4% vs. 32.2%) and subsequent hospitalizations (35.6% vs. 21.5%).

In recent years, SGLT2i has emerged as a novel drug class, known for its cardiac and renal protective effects. SGLT2is are a class of glucose-lowering agents, with substantial evidence supporting their unprecedented beneficial role in patients with HF, DM, or multiple cardiovascular risk factors [11,12,13]. The effect of SGLT2i may be achieved by modifying metabolic factors associated with increased AF risk, improving cardiac efficiency, or acting directly on cardiac ion channels [14].

While studies have suggested that SGLT2i reduces the occurrence of AF in HF and DM patients [15], concomitantly, there has been rapidly evolving research supporting its role in reducing AF recurrence after cardiac ablation in specific subgroups, mostly DM and HF patients [16,17]. However, the role of SGLT2i in AF recurrence after cardiac ablation in the general population remains unclear. This systematic review and meta-analysis aim to primarily evaluate the effect of SGLT2i on the recurrence of AF after catheter ablation in the general adult population compared with non-SGLT2i medications.

## 2. Methods

### 2.1. Study Design

This systematic review and meta-analysis included studies evaluating the impact of SGLT2i on AF recurrence following catheter ablation, compared to non-SGLT2i therapies. Studies meeting predefined eligibility criteria were included in the analysis. A thorough description of the study design is mentioned below.

### 2.2. Search Strategy

We searched four databases (PubMed, Scopus, Web of Science, and Cochrane) until 10th February 2025, with respective search criteria:

(1) PubMed and Cochrane: ((SGLT2 inhibitors or sodium-glucose cotransporter-2 inhibitors or SGLT2i or tofogliflozin or sotagliflozin or canagliflozin or dapagliflozin or empagliflozin or Bexagliflozin or Ertugliflozin) AND (atrial fibrillation or AFib)) AND (ablation), (2) Web of science: (“SGLT2 inhibitors” OR “sodium-glucose cotransporter-2 inhibitors” OR “SGLT2i” OR “tofogliflozin” OR “sotagliflozin” OR “canagliflozin” OR “dapagliflozin” OR “empagliflozin” OR “Bexagliflozin” OR “Ertugliflozin”) AND (“atrial fibrillation” OR “AFib”) AND ablation, (3) Scopus (“SGLT2 inhibitors” OR “sodium-glucose cotransporter-2 inhibitors” OR “SGLT2i” OR “tofogliflozin” OR “sotagliflozin” OR “canagliflozin” OR “dapagliflozin” OR “empagliflozin” OR “Bexagliflozin” OR “Ertugliflozin”) AND (“atrial fibrillation” OR “AFib”) AND ablation, and (4) Cochrane: (“SGLT2 inhibitors” OR “sodium-glucose cotransporter-2 inhibitors” OR “SGLT2i” OR “tofogliflozin” OR “sotagliflozin” OR “canagliflozin” OR “dapagliflozin” OR “empagliflozin” OR “Bexagliflozin” OR “Ertugliflozin”) AND (“atrial fibrillation” OR “AFib”) AND ablation.

### 2.3. Eligibility Criteria

We conducted title and abstract screening using predefined inclusion and exclusion criteria. Studies were included if they met the following conditions: (1) involved participants aged 18 years or older with a history of atrial fibrillation who were either already on, or initiated on, an SGLT2i at the time of or before ablation, regardless of DM status, and compared outcomes to those not on SGLT2i; (2) included subjects who had undergone any type of atrial fibrillation ablation; and (3) provided at least one year of follow-up. Studies were excluded if they (1) were published in a language other than English, (2) included participants younger than 18 years, or (3) did not report outcomes. All studies meeting these criteria proceeded to full-text screening, and original studies with at least one common outcome of interest were included.

### 2.4. Study Selection

A total of 270 studies were imported into Rayyan, an online review management platform (https://www.rayyan.ai/). After removing 64 duplicate records, 206 unique studies remained for screening. Title and abstract screening were conducted independently by three blinded reviewers: all 206 studies were screened by the first reviewer, the first 103 by the second, and the remaining 103 by the third, and conflicts were resolved by a fourth reviewer. This process resulted in 13 studies for full-text review. After full-text screening, studies that were not original research and had no common outcomes were excluded, resulting in a total of 9 studies being obtained. Out of these, 7 were cross-sectional studies, and 2 were randomized controlled trials (RCTs), which were used for the final systematic review and meta-analysis.

### 2.5. Data Extraction

Baseline and study characteristics of the included studies were extracted by two authors. Baseline characteristics includes (1) study arms and number of participants, (2) demographics: age, sex, body mass index (BMI), (3) type of AF: persistent or paroxysmal, (4) comorbidities: hypertension, diabetes mellitus, heart failure, coronary artery disease, hyperlipidemia, renal and thyroid disease, (5) echocardiography properties: ejection fraction, LAD, (6) medications: anti-arrhythmic drugs, diuretics, metoprolol, metformin, GLP1 and DPP4, (7) type of ablation: radiofrequency, cryoablation. Study characteristics include country, design, sample size, study period, inclusion criteria, blanking period, definition of AF recurrence, and follow-up schedule.

#### Definition of AF Recurrence

AF recurrence was defined as atrial fibrillation lasting for >30 s without concomitant use of antiarrhythmic drugs after the three-month blanking period. This definition was used across all studies.

### 2.6. Statistical Analysis

Odds ratio (OR) was used to calculate the statistical analysis of the pooled data for AF recurrence at all follow-ups. Hazardous ratio (HR) was used to calculate the multivariate AF recurrence risk and change in LAD as the pooled data was obtained in HR form. A random-effect model was employed for all pooled statistical analyses, using the Mantel-Haenszel statistical method to determine effect sizes and assess heterogeneity among the studies. Study heterogeneity was calculated by Tau^2^, Chi^2^, and I^2^ statistics, with between-study variance estimated by DerSimonian and Laird method and confidence Interval (CI) was calculated by the Hartung-Knapp-Sidik-Jonkman method, which were applied in the sequence according to the Cochrane handbook. Sensitivity analysis was performed by sequentially excluding each study to assess its impact on the pooled estimates and to evaluate changes in heterogeneity. All statistical, heterogeneity, and sensitivity analyses were conducted using Review Manager (RevMan), Version 7.2.0 (Edition 7.12.0).

### 2.7. Risk of Bias

Risk of bias (ROB) was evaluated by two authors, with blind on and the third author assisted with resolving conflicts. The National Institutes of Health (NIH) ROB and Cochrane ROB2 tools were used for quality assessment of retrospective cohort studies and RCTs, respectively.

### 2.8. Publication Bias

Publication bias was assessed using Egger’s regression test and funnel plot analysis. Egger’s regression test and funnel plots were constructed for both primary and secondary outcomes.

### 2.9. Registration

This systematic review and meta-analysis were prospectively registered in the PROSPERO database (Registration ID: CRD420251069826).

## 3. Results

### 3.1. Literature Search

**Figure 1:**
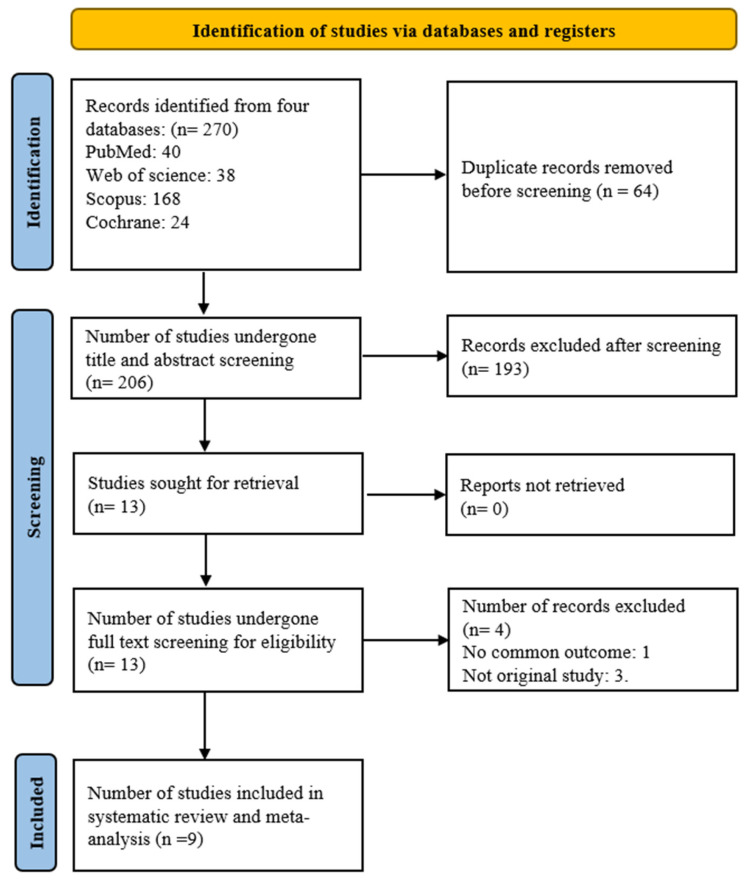
PRISMA 2020 flow chart diagram [25].

Out of 270 studies, after removing 64 duplicates, 193 were excluded based on title and abstract screening. Thirteen studies underwent full-text screening, of which three were poster presentations and one reported a different outcome from others. Ultimately, nine studies (seven cross-sectional and two RCTs) were included in the meta-analysis [16,17,18,19,20,21,22,23,24]. The study selection process is detailed in the PRISMA 2020 flow chart diagram presented in Figure 1 [25].

### 3.2. Baseline Characteristics

Table 1 summarizes the key characteristics of the included studies, while Table 2 presents the baseline characteristics of the study populations. A total of 6874 participants (non-SGLT2i, N = 3693; SGLT2, N = 3181) were included across the studies. The demographic profile showed a mean age of 64.6 years, with 70.7% of the population being male. Common comorbidities included hypertension (84.5%) and DM (83.9%). At baseline, 68% of participants were on anti-arrhythmic drugs, and 78.5% were receiving beta-blockers. All patients underwent pulmonary vein isolation as the ablation strategy (100%). Not all baseline characteristics were available for pooled analysis, as some studies did not report certain variables.

### 3.3. Risk of Bias

**Figure 2:**
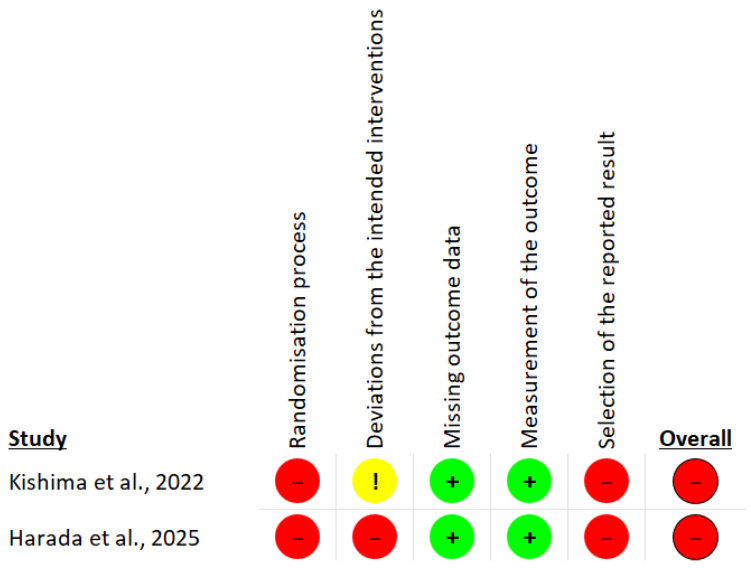
Visual representation of the overall and domain-specific risk of bias for the two included randomized controlled trials [20,21]. Red/−: High Risk, Green/+: Low risk, Yellow/!: Some concerns.

As summarized in Table 3, the NIH ROB tool was used to assess the quality of the retrospective studies included in this review. None of the studies reported whether outcome assessors were blinded to participants’ exposure status. Furthermore, all studies lacked details regarding sample size justification, power calculations, or estimates of variance and effect size. Except for the study by Qi et al., no information was provided on exposure levels or follow-up assessments. Additionally, ROB assessments for the two RCTs, Kishima et al. and Harada et al., indicate an overall high risk. Both studies exhibited high risk in the domains of the randomization process and selection of the reported results. For deviations from intended interventions, Kishima et al. was rated as having some concerns, whereas Harada et al. was assessed as high risk. In contrast, both studies demonstrated low risk in the domains of missing outcome data and outcome measurement. Detailed signaling questions and corresponding responses are summarized in Table 4, with a visual representation provided in Figure 2.

### 3.4. Clinical Outcomes Analysis

#### 3.4.1. Analysis of the Total Number of Subjects with AF Recurrence by the Final Follow-Up Post-Ablation

A total of 3181 subjects were included in the SGLT2i group and 3693 in the non-SGLT2i group. The duration of final follow-up varied across the included studies: four studies (Abu-Qaoud et al., Kishima et al., Harada et al., and Qi et al.) reported outcomes at 12 months, while other studies’ periods were of 18 months (Noh et al.), 24 months (Hakgor et al.), 36 months (Luo et al.), 48 months (Zhao et al.), and 80 months (Liu et al.).

**Figure 3:**
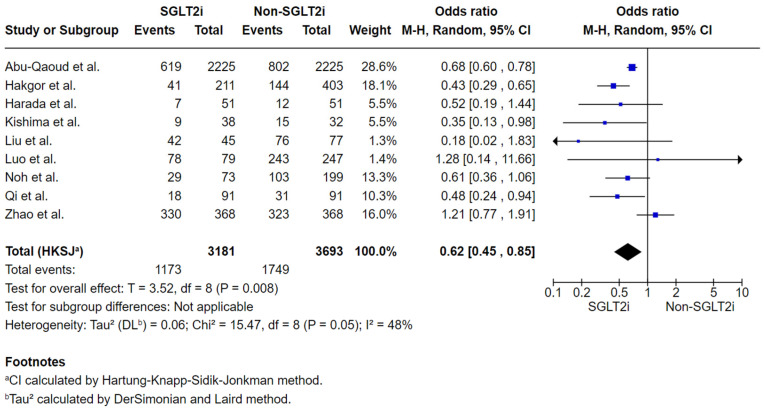
Statistical analysis and forest plot illustrates the odds ratio of AF recurrence by the final follow-up of post-ablation in subjects receiving Sodium-glucose cotransporter 2 inhibitor (SGLT2i) vs non- SGLT2i across studies. Squares represent individual study estimates, sized by study weight; horizontal lines show 95% confidence interval (CI). The diamond denotes the pooled effect estimate and its 95% CI [16,17,18,19,20,21,22,23,24].

AF recurrence was observed in 1173 participants in the SGLT2i group and 1749 in the non-SGLT2i group, representing a reduction in AF recurrence in the SGLT2i group compared to the non-SGLT2i group. This difference was statistically significant (OR: 0.62; 95% CI: 0.45–0.85; *p* = 0.008; Figure 3), with moderate heterogeneity among studies (*p* = 0.05; I^2^ = 48%). The moderate heterogeneity detected might be attributed to differences in the follow-up durations across the studies included in this outcome analysis, which possibly have influenced the effect estimates and contributed to variability in the pooled results. The sensitivity analysis excluding Zhao et al. yielded consistent findings demonstrating reduced AF recurrence in the SGLT2i group (OR: 0.58, 95% CI: 0.47–0.72, *p* = 0.0006, Figure S1), with low heterogeneity (*p* = 0.29, I^2^ = 18%). A possible explanation for the reduced heterogeneity observed in the sensitivity analysis after excluding Zhao et al. is that this study’s outcome results differed disproportionately from those of the other included studies. The same effect was not observed when excluding the work of Luo et al., despite its similarly divergent results, likely because Zhao et al. had a substantially larger sample size, thereby exerting a greater influence on the pooled estimates.

#### 3.4.2. Secondary Outcomes

##### Analysis of Total Number of Subjects with AF Recurrence by the First Follow-Up Within 12 to 24 Months Post-Ablation

**Figure 4:**
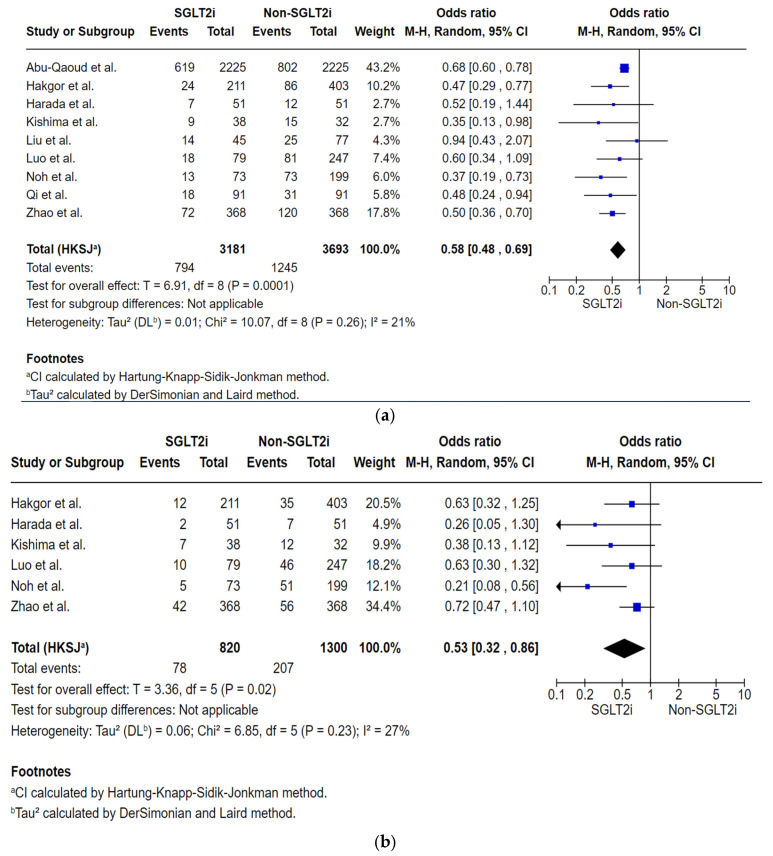

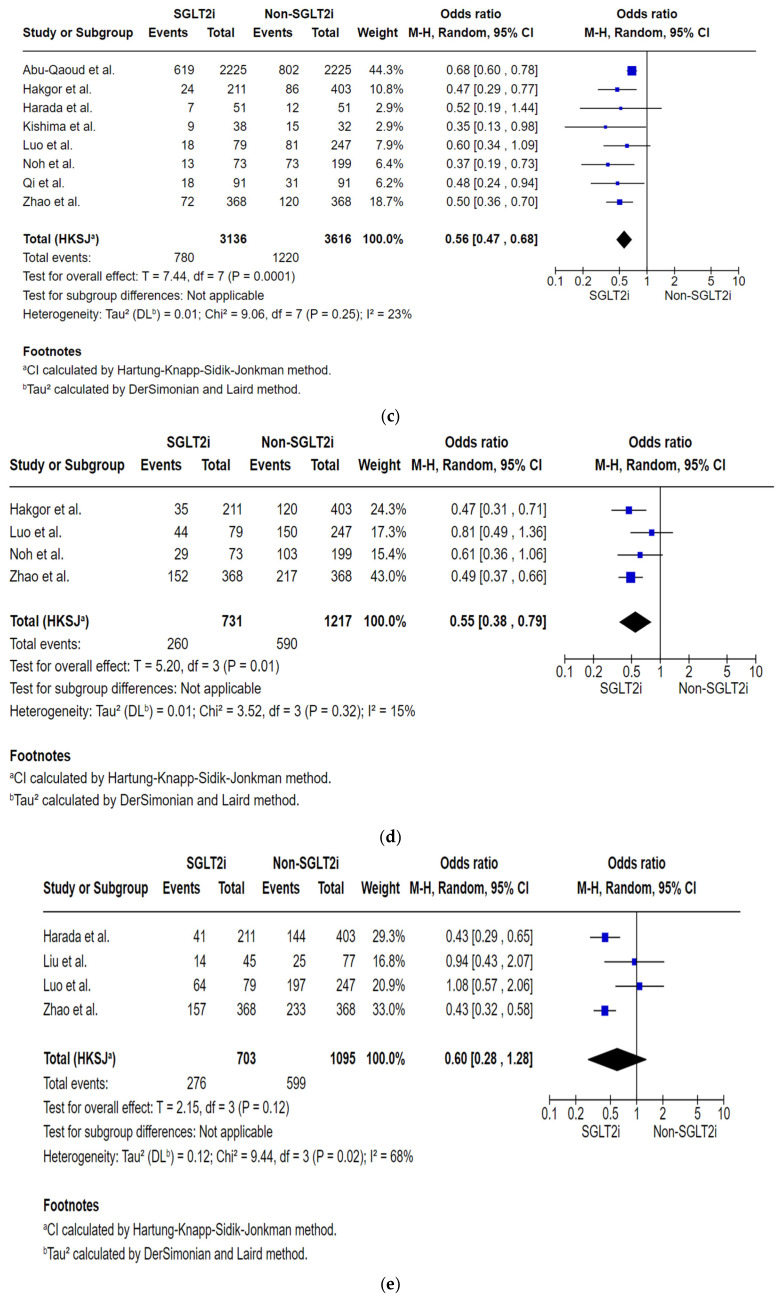

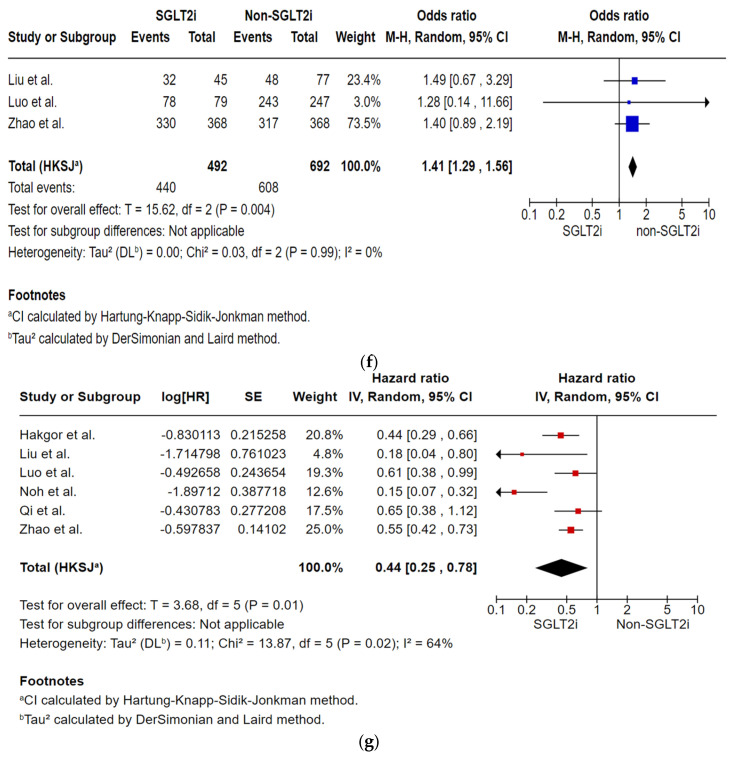
(**a**) Statistical analysis and forest plot illustrates the odds ratio of AF recurrence by the first follow-up within 12 to 24 months post-ablation in subjects receiving Sodium-glucose cotransporter 2 inhibitor (SGLT2i) vs non- SGLT2i across studies [16,17,18,19,20,21,22,23,24]. (**b**) Statistical analysis and forest plot illustrates the odds ratio of AF recurrence by the 6-month follow-up post-ablation in subjects receiving Sodium-glucose cotransporter 2 inhibitor (SGLT2i) vs non- SGLT2i across studies [17,18,19,20,21,24]. (**c**) Statistical analysis and forest plot illustrates the odds ratio of AF recurrence by the 12-month follow-up post-ablation in subjects receiving Sodium-glucose cotransporter 2 inhibitor (SGLT2i) vs non- SGLT2i across studies [16,17,18,19,20,21,23,24]. (**d**) Statistical analysis and forest plot illustrates the odds ratio of AF recurrence by the 18-month follow-up post-ablation in subjects receiving Sodium-glucose cotransporter 2 inhibitor (SGLT2i) vs non- SGLT2i across studies [17,18,19,24]. (**e**) Statistical analysis and forest plot illustrates the odds ratio of AF recurrence by the 24-month follow-up post-ablation in subjects receiving Sodium-glucose cotransporter 2 inhibitor (SGLT2i) vs non- SGLT2i across studies [17,18,21,22]. (**f**) Statistical analysis and forest plot illustrates the odds ratio of AF recurrence by the 36 to 42- month follow-up post-ablation in subjects receiving Sodium-glucose cotransporter 2 inhibitor (SGLT2i) vs non- SGLT2i across studies CI [17,18,22]. (**g**) Statistical analysis and forest plot illustrates the odds ratio of multivariate risk of AF recurrence in subjects receiving Sodium-glucose cotransporter 2 inhibitor (SGLT2i) vs non- SGLT2i across studies [17,18,19,22,23,24]. Squares represent individual study estimates, sized by study weight; horizontal lines show 95% confidence interval (CI). The diamond denotes the pooled effect estimate and its 95% CI.

In this analysis also 3181 subjects were included in the SGLT2i group, and 3693 in the non-SGLT2i group. The reported outcomes are based on pooled data assessing AF recurrences by the 12-month follow-up across included studies, except for Liu et al., which reported AF recurrences by the first follow-up, starting at 20 months post-ablation. AF recurrence occurred in 794 subjects in the SGLT2i group and 1245 in the non-SGLT2i group, indicating lower AF recurrence in the SGLT2i group compared to the non-SGLT2i group (OR: 0.58, 95% CI: 0.48–0.69, *p* = 0.0001, Figure 4a), with low heterogeneity (*p* = 0.26, I^2^ = 21%). The sensitivity analysis excluding Abu-Qaoud et al. yielded consistent findings demonstrating lower AF recurrence in the SGLT2i group (OR: 0.51, 95% CI: 0.42–0.61, *p* < 0.0001, Figure S2), with no observed heterogeneity (*p* = 0.77; I^2^ = 0%). The reduced heterogeneity observed in the sensitivity analysis following the exclusion of Abu-Qaoud et al. may be attributable to its disproportionately large sample size relative to the other included studies.

##### Analysis of the Total Number of Subjects with AF Recurrence by the Specific Follow-Up Intervals

The outcome data were pooled from the included studies that reported AF recurrence by the specific follow-up intervals. AF recurrences by the 6-month follow-up, 78 out of 820 in the SGLT2i group, 207 out of 1300 in the non-SGLT2i group, demonstrate a decrease in AF recurrence in the SGLT2i group (OR: 0.53, 95% CI: 0.32–0.86, *p* = 0.02, I^2^ = 27%, Figure 4b). By the 12-month follow-up, 780 out of 3136 in the SGLT2i group, 1220 out of 3616 in the non-SGLT2i group, demonstrate a decrease in AF recurrence in the SGLT2i group (OR: 0.56, 95% CI: 0.47–0.68, *p* = 0.0001, I^2^ = 23%, Figure 4c). By the 18-month follow-up, 260 out of 731 in the SGLT2i group, 590 out of 1217 in the non-SGLT2i group, demonstrated a decrease in AF recurrence in the SGLT2i group (OR: 0.55, 95% CI: 0.38–0.79, *p* = 0.01, I^2^ = 15%, Figure 4d). All results demonstrated statistically significant reductions in AF recurrence associated with SGLT2i therapy.

AF recurrence by the 24-month follow-up were observed in 276 of 703 patients in the SGLT2i group and 599 of 1095 patients in the non-SGLT2i group, reflecting a reduction in AF recurrence in the SGLT2i group compared to non-SGLT2i group; however, this difference did not reach statistical significance (OR: 0.60, 95% CI: 0.28–1.28, *p* = 0.12, I^2^ = 68%, Figure 4e). All the above outcomes resulted in similar findings after sensitivity analysis, as demonstrated in Figures S3–S6. In contrast to the earlier findings, by the 36- to 42-month follow-up period, AF recurrence was observed in 440 of 492 subjects in the SGLT2i group and 608 of 692 subjects in the non-SGLT2i group, representing a higher recurrence in the SGLT2i group compared to non-SGLT2i group. This difference was found to be statistically significant (OR: 1.41, 95% CI: 1.29–1.56, *p* = 0.004, I^2^ = 0%, Figure 4f).

##### Analysis of Multivariate Risk of AF Recurrence

Multivariable Cox regression analyses were conducted to evaluate the risk of atrial fibrillation (AF) recurrence following ablation while accounting for various covariates, including the use of SGLT2 inhibitors (SGLT2i). Hazard ratios (HRs) and 95% CIs were extracted from each study and pooled to derive a combined estimate. Overall, SGLT2i use was associated with a 56% reduction in the risk of AF recurrence compared to the non-SGLT2i group (HR: 0.44; 95% CI: 0.25–0.78; *p* = 0.02; I^2^ = 64%; Figure 4g) and similar results were observed after sensitivity analysis (HR: 0.53, 95% CI: 0.41–0.70, *p* = 0.003, I^2^ = 0%, Figure S7). Hakgor et al. and Luo et al. each published two multivariate analysis models. We incorporated the values from all of these models, resulting in three additional analyses that produced consistent results (Figures S8–S10).

##### Analysis of Left Atrial Diameter Change

The change in LAD from the two studies was reported as hazard ratios with 95% CI and was subsequently pooled to derive a combined estimate. The use of SGLT2i was significantly associated with a reduction in LAD among patients with a baseline LAD of ≥45 mm (OR = 0.52; 95% CI: 0.41–0.66; *p* = 0.02, I^2^ = 0%, Figure S11). In contrast, no statistically significant change in LAD was observed in patients with a baseline LAD < 45 mm (OR = 0.49; 95% CI: 0.01–17.59; *p* = 0.24, I^2^ = 57%, Figure S12).

### 3.5. Univariate Meta-Regression Model for Primary Outcome

To investigate potential sources of heterogeneity in AF recurrence by the final follow-up post-ablation, we conducted univariate meta-regression analyses using a random-effect model. The dependent variable was the log-odds ratio of AF recurrence by the final follow-up in each study. Each study-level covariate was analyzed separately, including patient demographics and baseline clinical characteristics. The covariates assessed were mean age, promotion of male patients, BMI, left ventricular ejection fraction (LVEF), LAD, and proportions of hypertension, DM, HTN, CAD, as well as prior use of AAD or beta-blocker therapy.

Among the tested variables, only LAD was significantly associated with AF recurrence (coefficient: 0.176; 95% CI: 0.049–0.304; *p* = 0.007), suggesting that LAD may predict a higher risk of recurrence. Other covariates, including age (*p* = 0.81), BMI (*p* = 0.6), and LVEF (*p* = 0.21), did not show significant associations, as presented in Table 5. These results suggest that structural remodeling, particularly atrial enlargement, may play a more critical role in post-ablation outcomes than demographic or comorbidity profiles alone. Visual presentations of the univariate meta-regression analyses for the individual variables are shown in Figures S13–S23.

### 3.6. Publication Bias

**Figure 5:**
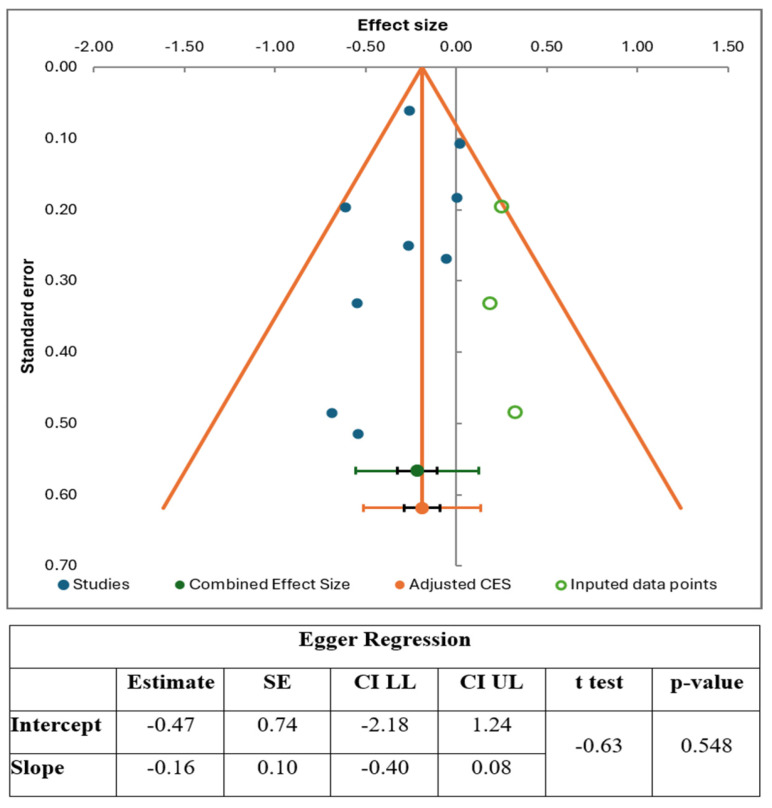
The Funnel Plot with Eggers test depicting the publication bias of AF recurrence in subjects by the final follow-up.

Egger’s regression test and funnel plot are illustrated in Figure 5. The funnel boundaries formed by the orange diagonal lines represent the pseudo–95% confidence intervals, demonstrating the expected range of study estimates around the pooled effect size in the absence of bias. The studies around the pooled effect size revealed a degree of asymmetry, with an apparent deficit of studies on the right side, raising the possibility of small-study effects or publication bias. To address this, the trim-and-fill method was applied, resulting in three imputed studies represented by green open circles on the right side of the plot. These additions slightly adjusted the overall effect size to the right, as indicated by the orange circle (adjusted combined effect size), compared to the original combined effect size shown by the green dot. Egger’s regression test revealed an intercept estimate of −0.47 (SE = 0.74), with a 95% confidence interval ranging from −2.18 to 1.24 and a *t*-test value of −0.63 (*p* = 0.548), indicating no statistically significant evidence of funnel plot asymmetry or publication bias. The slope was estimated at −0.16 (SE = 0.10) with a 95% CI of −0.40 to 0.08; although slightly negative, the confidence interval included zero, suggesting no statistically significant small-study effects.

Despite the visual asymmetry, the non-significant results of Egger’s regression test and the trim-and-fill adjustment suggest no strong evidence of publication bias in the included studies. Egger’s regression test generally requires at least 10 studies to yield a reliable interpretation; therefore, we cannot rule out the possibility of publication bias, as suggested by the asymmetry observed in the funnel plots. The funnel plots and Egger’s test analyses for other AF recurrence outcomes and multivariate AF recurrence risk are presented in Figures S24–S30. Funnel plots for LAD outcomes are also depicted; however, Egger’s test could not be performed for this outcome due to the inclusion of only two studies (Figures S31 and S32).

### 3.7. Certainty Assessment Among Outcomes

The certainty assessment across the outcomes with AF recurrence in this meta-analysis was rated as very low according to GRADEpro criteria, mostly due to the inclusion of non-randomized studies, which introduced a serious risk of bias. While inconsistency, indirectness, and imprecision were generally not considered serious, several outcomes exhibited potential publication bias, primarily those involving follow-ups by the 6, 12, and 18 months post-ablation, as shown in Table 6. Additionally, the limited number of studies and small sample sizes in certain follow-up intervals further reduced confidence in the findings.

## 4. Discussion

In this meta-analysis, we demonstrated that the use of SGLT2 inhibitors is associated with a lower risk of AF recurrence after an index catheter ablation in an adult population, an effect that persisted up to 18 months of follow-up. Notably, SGLT2i use was also associated with a reduction in the size of LAD in patients with baseline LAD of ≥45 mm. By their beneficial role in reducing the AF recurrence after cardiac ablation, we hypothesize that SGLT2i would lower the need for redo ablation, cardioversion, or the need for use of AADs.

The principal pathophysiologic mechanism for AF recurrence remains electrical re-conduction between the left atrium and pulmonary veins [7]. A study showed that the use of AADs was actually associated with an increased risk of AF-related hospitalization, proving their limited role. As such, their role remains limited to select subgroups of patients having frequent AF recurrences [26]. RAAFT-2 trial showed superiority of ablation over AADs in reducing recurrence at 2 years (54.5% vs. 72%), yet in both groups rate of recurrence was high [27]. Several studies on the efficacy of CA in AF have demonstrated that the success rates drop within the first year and continue to decline during the subsequent years of follow-up [7]. The same trend was seen in both initial (single) and multiple recurrent ablations. From available evidence, the long-term efficacy of cardiac ablation in isolation remains unclear.

An RCT had previously demonstrated that treatment with AADs during the first 6 weeks after CA reduced the incidence of early recurrence, but their use did not prevent recurrence at 6 months [28]. Additionally, A meta-analysis of RCTs demonstrated that the short-term use of AADs after CA reduces the incidence of early recurrent atrial tachyarrhythmias but did not prevent late recurrence of AF (>3 months). These results emphasize a limited role for AADs in AF recurrence after CA [29]. The results from the ORBIT-AF registry showed that, compared to non-hospitalized patients, hospitalized AF patients were more likely to have concomitant heart failure and a higher mean CHADS2 score [30]. The most extensive systematic review and meta-analysis to date (75,279 participants) showed that SGLT2i may reduce the incidence or recurrence of AF in DM patients and showed a reduction in HF hospitalization or cardiovascular death in patients with DM, both with or without AF [11]. Given their proven benefits in HF and DM patients, SGLT2i may help in reducing AF recurrence in patients who have had ablation. Additionally, the monthly cost of SGLT2i ranges from approximately $0 to $50, depending on the patient’s insurance coverage [31]. This relatively low expense could lead to significant overall healthcare savings by reducing hospitalizations related to AF recurrence or associated complications.

The mechanisms by which SGLT2i exert their cardioprotective benefits are not clearly understood. SGLT2i exerts its beneficial effects through a multipronged approach. SGLT2i lowers blood pressure by inhibiting SGLT2 in the proximal convoluted tubule, leading to increased urinary excretion of sodium and glucose, which causes osmotic diuresis. Additionally, increased sodium delivery to the distal convoluted tubule suppresses renin release, subsequently reducing levels of angiotensin II and aldosterone, thereby contributing to blood pressure reduction [32]. SGLT2i promote weight loss primarily by enhancing lipid metabolism for energy production and reducing insulin secretion; these mechanisms are driven by the glucose-deprived state resulting from glucosuria [33]. This weight-reducing effect contributes to possible improvements in obstructive sleep apnea [34]. SGLT2i increases ketone production due to its hypoglycemic effect, which in turn causes organs like the kidneys and myocardium to use ketones for energy production, which generates the same energy as glucose with less oxygen requirement, thereby decreasing oxidative stress and increasing efficiency [35]. In addition, SGLT2i improve cardiac function by attenuating transforming growth factor (TGF)-beta induced fibroblast activation, reducing myocardial fibrosis and remodeling [36]. SGLT2i may exert anti-arrhythmic effects through sodium and calcium homeostasis within cardiomyocytes by inhibiting sodium-hydrogen exchanger (NHE), enhancing the sodium-calcium exchanger (NCX) activity, and increasing Sarco/Endoplasmic Reticulum Calcium ATPase (SERCA) expression [37]. Also, anti-inflammatory effect of SGLT2i is by modulating adenosine monophosphate-activated protein kinase (AMPK)/sirtuin-1 (SIRT1)/peroxisome proliferator-activated receptor-gamma coactivator 1α (PGC-1α) signaling pathways, suppressing mammalian target of rapamycin (mTOR) activity, inhibiting nod-like receptor family pyrin domain containing inflammasome platform 3 (NLRP3) inflammasome activation, and reducing pro-inflammatory cytokines such as IL-6, IL-1β, and TNF-α [38].

Recently published systematic reviews have focused on specific subgroups of patients, like T2DM, and had a follow-up period of up to 12 months. Zhao et al. [1]. and Abdelhadi et al. [39]., studied the effects of SGLT2i on AF recurrence after cardiac ablation, just like our study. However, they restricted their study to only the DM patient population. Moreover, a meta-analysis by Zhao et al. [1]., evaluated AF recurrence during the 18-month follow-up period. Our results align with those of this study. The results unique to this study were that patients with larger BMI, persistent AF, and longer AF duration seem to benefit more from SGLT2i therapy. However, this study was also restricted to the DM population, limiting generalization of the results to other patient populations. Additionally, a meta-analysis by Abdelhadi et al. [39]. showed a significant reduction in AF recurrence in patients receiving SGLT2i, explicitly focused on the DM population and without particular mention of the follow-up period. It was also associated with decreased all-cause hospitalizations, but no significant difference in all-cause mortality. This study also has some additional limitations, which were addressed to the editor [40]. To our knowledge, our study is the most comprehensive systematic review looking into the role of SGLT2i on AF recurrence after CA in the general adult population, including but not limited to patients with HF and DM, and has a longer follow-up period (up to 42 months).

One unexpected finding in our study was an increased risk of AF recurrence associated with the use of SGLT2i at 36–42 months of follow-up after catheter ablation. Currently, there is not much data on the long-term follow-up with SGLT2i. Findings in our study were based on limited studies, restricting their generalizability. A few potential explanations could be that SGLT2i has a limited potential role in reversing atrial fibrosis, which can progress slowly over time, potentially leading to AF recurrence. Alternatively, it could also be due to the progression of comorbidities like HF, DM, or CKD, which are not well-adjusted in the observational studies. These findings support the need for a multi-center RCT looking into long-term follow-ups.

Studies have shown that increasing LAD is associated with atrial fibrosis, which may lead to nonuniform anisotropy and heterogeneity in conduction [41]. A study carried out by Pappone et al. showed that LAD > 45 mm predicted a higher risk of recurrence of AF [42]. Another study supported this finding by showing larger LAD was a significant predictor of AF recurrence and AF progression after initial CA. The same study also showed that patients with lower LAD did not develop AF progression after initial CA [7]. The results from our study show that SGLT2i use was associated with a reduction in the size of LAD. The pooled analysis from two studies in our systematic review shows a statistically significant impact of SGLT2i on the reduction in LAD in patients with baseline LAD of ≥45 mm, which remains an independent risk factor for AF recurrence and progression after initial CA.

Our study was not confined to specific subgroups like other systematic reviews, which focused on patients with DM or HF, implying the results from this study could apply to the general population. While a meta-analysis based on RCTs would provide the highest data quality, significant innovations are underway in treatment modalities, such as catheter ablation, making a case for including case series and observational studies, as in our analysis. Our study is unique given its longer follow-up period (up to 42 months), which can have clinical implications and provide valuable insights while designing future prospective trials. Observational studies (seven in our study) may be subject to confounding. There may have been a few confounding factors that were not accounted for during the analysis of the outcomes. The differences in reporting by studies prevented us from performing subgroup analysis. Additionally, we conducted a univariate meta-regression analysis despite including only nine studies, which represents a limitation due to the potential for overfitting and reduced statistical power. Comprehensive data on the proportion of patients with different types of AF (paroxysmal, persistent, and permanent) were not available, which restricted the analysis of AF recurrence by these subgroups. Moreover, further studies are needed to evaluate the comparative efficiency of individual drugs within the SGLT2 inhibitor class, similar to the relative trials conducted in patients with heart failure [43].

## 5. Conclusions

Our study results suggest that SGLT2is are associated with a significant reduction in AF recurrence risk following catheter ablation, particularly within the 12- to 18-month post-ablation period. This effect remains significant even after accounting for other clinical variables, as supported by our analysis of the multivariate risk of AF recurrence. Reduction in AF recurrence in the SGLT2i group would mean a lesser need for redo ablation and use of AADs that are commonly associated with serious side effects. Furthermore, SGLT2i therapy appears to favorably contribute to structural remodeling, as evidenced by improvement in left atrial diameter after catheter ablation. Although the results are promising, prospective studies including multi-center RCTs are required to validate these findings in patients with AF who have undergone catheter ablation.

## Figures and Tables

**Table 1:** Study characteristics of included studies. T2DM: Type 2 Diabetes Mellitus, AF: Atrial fibrillation, AAD: Anti-arrhythmic drugs, ECG: Electrocardiography, RCT: Randomized control studies, HF: Heart failure, NT-proBNP: N-terminal pro-B-type natriuretic peptide, SGLT2i: Sodium glucose co-transporter 2 inhibitors, DPP4i: Dipeptidyl peptidase-4 inhibitors, CA: Catheter ablation.

Study	Site	Study Design	Total Sample Size	Study Period	Inclusion Criteria	Blanking Period	AF Recurrence Is Defined as	Follow-Up Schedule
Abu-Qaoud et al. (2023) [16]	USA	Retrospective cohort study	4450	1 April 2014 to 30 November 2021	Subjects who were >= 18 years of age, History of T2DM who had undergone AF ablation. Propensity score matching	3 months	Subject presented for cardioversion, new AAD class I or III, and re-do ablation in 12-month follow-up.	Subject presented for cardioversion, new AAD class I or III, and re-do ablation in 3 to 12 months of follow-up after ablation.
Hakgor et al. (2024) [24]	Turkey	Retrospective cohort study	614	2014 to 2021	Subjects who received catheter ablation for symptomatic AF.	3 months	AF lasting more than 30 s, either on ECG at follow-ups when symptomatic or 48 h Holter monitoring biannually.	Subjects had an ECG at 1, 3, 6, 12, 18, and 24 months. Also, had an ECG on the same day anytime when subjects had symptoms. Additionally, 48 h ambulatory Holter monitor was done biannually.
Harada et al. (2025) [21]	Japan	Prospective, RCT	102	June 2022 to November 2023	Subjects with persistent AF, symptoms/history/findings that suspect HF, and NT-proBNP >=400 pg/mL.	3 months	Any atrial tachy-arrhythmia lasting >30 s on 24-h Holter ECG monitoring at 6-month follow-up and 7-day Holter ECG monitoring at 12 months follow-up.	Subjects had 12-lead ESG at the cardiologist’s office at 1, 3, 6, 9, and 12 months after ablation. 24-h Holter monitoring was done at 6 months, and 7-day Holter monitoring was done at 12-month follow-up.
Kishima et al. (2022) [20]	Japan	Prospective, RCT	70	April 2017 and March 2020	Subjects with age >20 and <80 years, T2DM with non-valvular AF who underwent catheter ablation, and all subjects on oral anti-coagulant, duration of AF <1 year, not taking SGLT2i and/or DPP4i for >2 weeks before enrollment.	3 months	Episode of atrial tachyarrhythmia lasting for >30 s on Holter monitor.	Subjects followed up at the hospital at 1, 3, 6, 9, and 12 months after the CA or were referred to the emergency department for arrhythmia symptoms in between scheduled visits.
Liu et al. (2023) [22]	Taiwan	Retrospective cohort study	122	Jan 2016 and December 2021	Subjects with T2DM undergoing CA for anti-arrhythmic refractory AF.	3 months	Atrial tachyarrhythmia lasting longer than 30 s on a 12-lead ECG, Holter monitoring, or pacemaker/implantable cardioverter-defibrillator interrogation	Subjects had a 12-lead ECG and a 24-h Holter monitor at follow-ups at 1 week, 1 month, 3 months, 6 months, and every 3 to 6 months after CA or when subjects had symptoms.
Luo et al. (2022) [18]	China	Retrospective cohort study	326	January 2019 to February 2021	Subjects with T2DM and drug-refractory AF underwent radiofrequency CA	1 month	AF, atrial flutter or atrial tachycardia lasting for 30 s recorded on ECG or by 24-h Holter monitoring done at follow-ups or symptoms onset.	Subjects received outpatient or telephone follow-up at 1, 3, 6, 9, and 12 months, then every 6 months after ablation. Subjects had an ECG and a 24-h Holter monitor at each visit, and also when subjects had symptoms.
Noh et al. (2024) [19]	Korea	Retrospective cohort study	272	January 2018 to December 2022	Subjects included in this study underwent catheter ablation for symptomatic drug-refractory non-valvular AF.	3 months	AF, atrial tachycardia, or atypical atrial flutter lasting more than 30 s on a Holter monitor is done at follow-ups.	Subjects’ follow-ups are scheduled at 4 weeks, 3 months, 6 months, and every 3 to 4 months after that. Subjects on Holter monitor at 3 and 6 months and every 12 months after that.
Qi et al. (2024) [23]	China	Retrospective cohort study	182	December 2021 to January 2023	Subjects with age >= 18 years old, T2DM, non-valvular AF, 1st radiofrequency CA.	3 months	Any atrial tachyarrhythmia lasting for more than 30 s on ECG or Holter monitor during follow-up.	Subjects had 12-lead ECGs in the clinic at 1, 3, 6, and 12 months. Also, 24-h Holter monitoring every 6 months. Additionally, further Holter monitoring is done in the event of arrhythmic symptoms.
Zhao et al. (2024) [17]	China	Retrospective cohort study.	736	January 2017 to December 2022	Subjects with heart failure undergoing initial catheter ablation for AF. Propensity score matching.	3 months	Atrial tachyarrhythmia lasting more than 30 s on a 12-lead ECG or 24-h Holter monitoring.	Subjects followed up at clinics or by phone interview at the 3rd month, 6th month, and every 6 months thereafter.

**Table 2:** Baseline characteristics of subjects from the included studies. SD: Standard deviation, SGLT2i: Sodium glucose co-transporter 2 inhibitors, eGFR: Estimated glomerular filtration rate, CHA2DS2 VASc: Congestive heart failure, Hypertension, Age (≥75, doubled), Diabetes, Stroke/TIA (doubled), Vascular disease, Age (65–74), Sex category (Female), LVEF: Left ventricular ejection fraction, LAD: Left atrial diameter, HTN: Hypertension, T2DM: Type 2 diabetes mellitus, HF: Heart failure, COPD: Chronic obstructive pulmonary disease, CVA: Cerebrovascular accident, CAD: Coronary artery disease, HLD: Hyperlipidemia, AAD: Anti-arrhythmic drugs, GLP1: Glucagon-like peptide-1, DPP4i: Dipeptidyl peptidase-4 inhibitors, PVI: Pulmonary vein isolation, CTI: Cavo-tricuspid isthmus, SVCI: Superior vena cava isolation, RFCA: Radiofrequency catheter ablation.

Study	Abu-Qaoud et al. (2023) [16]		Hakgor et al. (2024) [24]		Harada et al. (2025) [21]		Kishima et al. (2022) [20]		Liu et al. (2023) [22]		Luo et al. (2022) [18]		Noh et al. (2024) [19]		Qi et al. (2024) [23]		Zhao et al. (2024) [17]	
Intervention	SGLT2i	Non-SGLT2i	SGLT2i	Non-SGLT2i	SGLT2i	Non-SGLT2i	SGLT2i	Non-SGLT2i	SGLT2i	Non-SGLT2i	SGLT2i	Non-SGLT2i	SGLT2i	Non-SGLT2i	SGLT2i	Non-SGLT2i	SGLT2i	Non-SGLT2i
Type of SGLT2i			Empagliflozin		Empaglifozin		Tofogliflozin	Anagliptin			Dapagliflozin		Dapagliflozin					
			Dapagliflozin		Dapagliflozin													
No of Participants in each Group	2225	2225	211	403	51	51	38	32	45	77	79	247	73	199	91	91	368	368
Age (years) Mean (SD)	65	65	59.8	57.1	70.2	72	70.3	70.3	60.1	63.2	63.4	63.8	73.47	71.72	70.9	67.3	63.5	62.7
Male (n)	1648	1642	129	226	33	38	26	22	35	54	48	144	61	175	52	48	242	239
BMI (kg/m^2^) Mean (SD)			27.5 (5.2)	26.3 (4.9)	25 (5.3)	24 (4.1)	25.5 (4.6)	25.3 (4.3)	28.6 (4.2)	27.3 (3.9)	25.9 (3.7)	26.7 (3.5)	25.16 (3.64)	23.73 (5.45)	26.1 (0.4)	26.5 (0.3)	26.4 (3.8)	26.4 (4.2)
Paroxysmal (n)			124	291			16	14	32	54			43	107				
Persistent (n)					51	51			13	23	35	119	30	92	91	91	279	291
AF duration (years) Mean (SD)					6.4 (11.4)	6.7 (5.6)			3.09 (3.1)	3.67 (3.73)					2.19 (0.38)	3.07 (0.46)		
Hemoglobin A1C (%) Mean (SD)			7.5 (2.5)	6.2 (1.9)	5.9 (0.4)	6 (0.4)	6.7 (0.6)	6.8 (0.9)	6.8 (0.7)	6.6 (0.8)	7.7 (1.4)	7.3 (1.2)			7.4 (0.1)	7.2 (0.1)		
Creatinine (mg/dL) Mean (SD)	1.2 (2.9)	1.3 (2.2)	1 (0.2)	0.9 (0.2)	0.9 (0.2)	0.9 (0.3)	0.83 (0.26)	0.75 (0.2)	1.01 (0.32)	0.97 (0.33)			1.25 (1.16)	1.1 (0.82)				
eGFR (mL/min) Mean (SD)			81.1 (24.3)	85.2 (25.1)	71 (26.7)	70.8 (29.5)	70.7 (22.8)	76.1 (21.1)	83.3 (24.1)	85.4 (29.3)	84.5 (17.3)	85 (18)	70.6 (23.02)	76.65 (25.08)			99 (28.6)	103.1 (27.7)
CHA2DS2 VASc Score Mean (SD)			3.1 (1.4)	1.9 (1.3)	3.4 (1.6)	3.3 (1.3)	2.6 (1.1)	2.7 (1.3)	2.8 (1.2)	2.9 (1.3)							3.4 (1.4)	3.5 (1.4)
LVEF (%) Mean (SD)			52.3 (13.5)	60.5 (8.2)	52.5 (11.8)	55.1 (7.4)	61.5 (11.3)	58 (15.4)	63.8 (11.7)	63.8 (10.7)	60.4 (6.7)	60 (7.2)	57.23 (7.26)	59.68 (6.18)	63.4 (0.7)	63.6 (0.6)	51.4 (10)	51.4 (11.6)
LAD (mm) Mean (SD)			41.6 (7.1)	40.5 (7.1)	42.5 (5.9)	42.9 (5.5)	45.6 (7.5)	44.7 (5)	44.2 (5.7)	43.5 (6.4)	40.2 (6.4)	41 (6.5)	43.94 (5.45)	45.35 (5.3)	41.9 (0.5)	40.8 (0.4)	45.8 (7.7)	45.1 (5.7)
HTN (n)	2062	2067	153	276	27	27	26	19	36	57	53	157	50	141	69	79	258	251
T2DM (n)	2225	2225	110	38	0	0	38	32	45	77	79	247	21	68	91	91	182	198
HF (n)	1316	1302	77	36	51	51	10	9	6	7					3	5	368	368
COPD (n)			26	44					0	2			13	23				
CVA (n)			21	25	5	6	6	9	0	9	9	31					53	49
CAD (n)	1480	1473	98	114			2	2	10	9	31	102	3	33			49	61
Smoking (n)			70	132					6	9	25	53						
Renal disease (n)	562	577													2	2	15	25
HLD (n)	1892	1907					26	19	26	47	21	50						
Thyroid disease (n)	677	666							7	9								
AAD (n)	2029	2008	71	112	5	2	4	6	27	63	17	58	73	198			0	0
CLASS IC (n)									14	32	7	14	0	17				
CLASS III (n)					1	0			13	31	10	44	73	178				
Beta-blocker (n)	2107	2110	158	241	44	38	21	15	32	47	32	87	3	26	29	30	197	178
Metformin (n)	1597	1642					8	5	36	54	42	139			51	46		
Insulin (n)	1635	1604	51	14			1	0	1	5	7	39			16	11		
Diuretics (n)	1824	1832	54	27	25	27	6	7	12	6	0	9	21	60	6	11	262	286
GLP1 (n)									1	3	1	2						
DPP4i (n)							0	32	12	38	7	20						
Cryo-ablation PVI (n)			184	361	10	7	8	10	45	77			42	56				
CTI ablation (n)							30	20	36	60			19	99				
SVCI (n)							8	4			14	43	5	9				
Linear ablation (n)									19	32	60	174					279	291
RFCA PVI (n)			27	42	41	44	30	22			79	247	31	143	91	91	368	368

**Table 3:** National Institute of Health Risk of bias of retrospective cohort studies. NA: Not applicable, NR: Not reported, CD: Cannot determine.

Criteria	Abu-Qaoud et al.	Liu et al.	Luo et al.	Qi et al.	Hakgor et al.	Noh et al.	Zhao et al.
Was the research question or objective in this paper clearly stated?	Yes	Yes	Yes	Yes	Yes	Yes	Yes
Was the study population clearly specified and defined?	Yes	Yes	Yes	Yes	Yes	yes	Yes
Was the participation rate of eligible persons at least 50%?	Yes	Yes	Yes	Yes	Yes	Yes	Yes
Were all the subjects selected or recruited from the same or similar populations (including the same time period)? Were inclusion and exclusion criteria for being in the study prespecified and applied uniformly to all participants?	Yes	Yes	Yes	Yes	Yes	Yes	Yes
Was a sample size justification, power description, or variance and effect estimates provided?	No	No	No	No	No	No	No
For the analyses in this paper, were the exposure(s) of interest measured prior to the outcome(s) being measured?	Yes	Yes	Yes	Yes	Yes	Yes	Yes
Was the timeframe sufficient so that one could reasonably expect to see an association between exposure and outcome if it existed?	Yes	Yes	yes	Yes	Yes	Yes	Yes
For exposures that can vary in amount or level, did the study examine different levels of the exposure as related to the outcome (e.g., categories of exposure, or exposure measured as continuous variable)?	No	No	No	Yes	No	No	No
Were the exposure measures (independent variables) clearly defined, valid, reliable, and implemented consistently across all study participants?	Yes	Yes	Yes	Yes	Yes	Yes	Yes
Was the exposure(s) assessed more than once over time?	NA	NR	NR	Yes	NR	NR	NR
Were the outcome measures (dependent variables) clearly defined, valid, reliable, and implemented consistently across all study participants?	No	Yes	Yes	Yes	Yes	Yes	Yes
Were the outcome assessors blinded to the exposure status of participants?	No	No	No	No	No	No	No
Was loss to follow-up after baseline 20% or less?	CD	Yes	Yes	Yes	Yes	Yes	Yes
Were key potential confounding variables measured and adjusted statistically for their impact on the relationship between exposure(s) and outcome(s)?	No	Yes	Yes	Yes	Yes	Yes	Yes

**Table 4:** Risk of bias of randomized control studies. Y: Yes, PY: Probably yes, PN: Probably no, N: No, NI: No information, NA: Not applicable.

Domain	Signaling Question	Kishima et al.	Harada et al.
**Bias arising from the randomization process**	1.1 Was the allocation sequence random?	Y	PN
	1.2 Was the allocation sequence concealed until participants were enrolled and assigned to interventions?	N	NI
	1.3 Did baseline differences between intervention groups suggest a problem with the randomization process?	N	PY
	**Risk of bias judgement**	**High**	**High**
**Bias due to deviations from intended interventions**	2.1.Were participants aware of their assigned intervention during the trial?	NI	NI
	2.2.Were carers and people delivering the interventions aware of participants’ assigned intervention during the trial?	Y	Y
	2.3. If Y/PY/NI to 2.1 or 2.2: Were there deviations from the intended intervention that arose because of the experimental context?	NI	NI
	2.4 If Y/PY to 2.3: Were these deviations likely to have affected the outcome?	NA	NA
	2.5. If Y/PY/NI to 2.4: Were these deviations from intended intervention balanced between groups?	NA	NA
	2.6 Was an appropriate analysis used to estimate the effect of assignment to intervention?	Y	NI
	2.7 If N/PN/NI to 2.6: Was there potential for a substantial impact (on the result) of the failure to analyse participants in the group to which they were randomized?	NA	NI
	**Risk of bias judgement**	**Some concerns**	**High**
**Bias due to missing outcome data**	3.1 Were data for this outcome available for all, or nearly all, participants randomized?	Y	Y
	3.2 If N/PN/NI to 3.1: Is there evidence that result was not biased by missing outcome data?	NA	NA
	3.3 If N/PN to 3.2: Could missingness in the outcome depend on its true value?	NA	NA
	3.4 If Y/PY/NI to 3.3: Is it likely that missingness in the outcome depended on its true value?	NA	NA
	**Risk of bias judgement**	**Low**	**Low**
**Bias in measurement of the outcome**	4.1 Was the method of measuring the outcome inappropriate?	N	N
	4.2 Could measurement or ascertainment of the outcome have differed between intervention groups?	N	N
	4.3 Were outcome assessors aware of the intervention received by study participants?	N	NI
	4.4 If Y/PY/NI to 4.3: Could assessment of the outcome have been influenced by knowledge of intervention received?	NA	PN
	4.5 If Y/PY/NI to 4.4: Is it likely that assessment of the outcome was influenced by knowledge of intervention received?	NA	NA
	**Risk of bias judgement**	**Low**	**Low**
**Bias in selection of the reported result**	5.1 Were the data that produced this result analysed in accordance with a pre-specified analysis plan that was finalized before unblinded outcome data were available for analysis?	Y	NI
	5.2 … multiple eligible outcome measurements (e.g., scales, definitions, time points) within the outcome domain?	Y	Y
	5.3 … multiple eligible analyses of the data?	Y	Y
	**Risk of bias judgement**	**High**	**High**
**Overall bias**	**Risk of bias judgement**	**High**	**High**

**Table 5:** Univariate meta-regression model for primary outcome. CI: Confidence interval, SE: Standard error, BMI: Body mass index, LVEF: left ventricular ejection fraction, LAD: Left atrial diameter, CAD: Coronary artery disease.

Covariate	Coefficient	Lower CI	Upper CI	SE	*p*-Value
Age	−0.007	−0.066	0.052	0.030	0.812
Male Proportion	0.000	−0.000	0.000	<0.001	0.278
BMI	−0.098	−0.466	0.269	0.188	0.600
LVEF (%)	−0.045	−0.117	0.026	0.036	0.213
LAD (mm)	0.176	0.049	0.304	0.065	0.007
Hypertension	0.000	−0.000	0.000	<0.001	0.293
Diabetes mellitus	0.000	−0.000	0.000	<0.001	0.319
Heart Failure	0.000	−0.000	0.000	<0.001	0.149
CAD	0.000	−0.000	0.001	<0.001	0.789
History of Antiarrhythmic Use	0.000	−0.000	0.000	<0.001	0.54
History of Beta Blocker Use	0.000	−0.000	0.000	<0.001	0.319

**Table 6:** Certainty assessment among the outcomes. The risk in the intervention group (and its 95% confidence interval) is based on the assumed risk in the comparison group and the relative effect of the intervention (and its 95% CI). AF: Atrial fibrillation, CI: Confidence interval, OR: Odds ratio.

Outcome № of Participants (Studies)	Relative Effect (95% CI)	Anticipated Absolute Effects (95% CI)			Certainty	What Happens
		Without SGLT2i	With SGLT2i	Difference		
AF recurrence by the final follow-up post-ablation № of participants: 6874 (9 non-randomised studies)	**OR 0.62** (0.45 to 0.85)	47.4%	**35.8%** (28.8 to 43.3)	**11.6% fewer** (18.5 fewer to 4 fewer)	⨁◯◯◯ Very low	Very low certainty due to the majority of studies included are observational studies with a serious risk of bias and inconsistency in results across studies.
AF recurrence by the first follow-up within 12 to 24 months post-ablation № of participants: 6874 (9 non-randomised studies)	**OR 0.58** (0.48 to 0.69)	33.7%	**22.8%** (19.6 to 26)	**10.9% fewer** (14.1 fewer to 7.7 fewer)	⨁◯◯◯ Very low	Very low certainty due to the majority of studies included are observational studies with a serious risk of bias, and publication bias is strongly suspected.
AF recurrences by the 6-month follow-up post-ablation № of participants: 2120 (6 non-randomised studies)	**OR 0.53** (0.32 to 0.86)	15.9%	**9.1%** (5.7 to 14)	**6.8% fewer** (10.2 fewer to 1.9 fewer)	⨁◯◯◯ Very low	Very low certainty due to the majority of studies included are observational studies with a serious risk of bias, and publication bias is strongly suspected.
AF recurrences by the 12-month follow-up post-ablation № of participants: 6752 (8 non-randomised studies)	**OR 0.56** (0.47 to 0.68)	33.7%	**22.2%** (19.3 to 25.7)	**11.6% fewer** (14.4 fewer to 8 fewer)	⨁◯◯◯ Very low	Very low certainty due to the majority of studies included are observational studies with a serious risk of bias, and publication bias is strongly suspected.
AF recurrences by the 18-month follow-up post-ablation № of participants: 1948 (4 non-randomised studies)	**OR 0.55** (0.38 to 0.79)	48.5%	**34.1%** (26.3 to 42.6)	**14.4% fewer** (22.1 fewer to 5.8 fewer)	⨁◯◯◯ Very low	Very low certainty due to the majority of studies included are observational studies with a serious risk of bias.
AF recurrences by the 24-month follow-up post-ablation № of participants: 1798 (4 non-randomised studies)	**OR 0.60** (0.28 to 1.28)	54.7%	**42.0%** (25.3 to 60.7)	**12.7% fewer** (29.4 fewer to 6 more)	⨁◯◯◯ Very low	Very low certainty due to the majority of studies included are observational studies with a serious risk of bias and inconsistency in results across studies.
AF recurrences by the 36 to 42-month follow-up post-ablation № of participants: 1184 (3 non-randomised studies)	**OR 1.41** (1.29 to 1.56)	87.9%	**91.1%** (90.3 to 91.9)	**3.2% more** (2.5 more to 4 more)	⨁◯◯◯ Very low	Very low certainty due to the majority of studies included are observational studies with a serious risk of bias.

## Data Availability

The data included in this study are either mentioned in this manuscript, in figures and tables, or in the Supplementary Materials.

## Supplementary Materials

The following supporting information can be downloaded at: https://www.mdpi.com/article/10.3390/jcm14228001/s1, PRISMA_2020_checklist; Figure S1: After sensitivity analysis, AF recurrence by the final follow-up post-ablation; Figure S2: After sensitivity analysis, AF recurrence by the first follow-up within 12 to 24 months post-ablation; Figure S3: After sensitivity analysis, AF recurrences by the 6-month follow-up; Figure S4: After sensitivity analysis, AF recurrences by the 12-month follow-up; Figure S5: After sensitivity analysis, AF recurrences by the 18-month follow-up; Figure S6: After sensitivity analysis, AF recurrences by the 24-month follow-up; Figure S7: After sensitivity analysis, 1st Multivariate risk of AF recurrence; Figure S8: Statistical analysis and forest plot of 2nd Multivariate risk of AF recurrence; Figure S9: Statistical analysis and forest plot of 3nd Multivariate risk of AF recurrence; Figure S10: Statistical analysis and forest plot of 4th Multivariate risk of AF recurrence; Figure S11: Forest plot of left atrial diameter (LAD) improvement in subjects with LAD ≥ 45 Mm; Figure S12: Forest plot of left atrial diameter (LAD) improvement in subjects with LAD < 45 mm; Figure S13: Univariate meta-regression analysis of Age; Figure S14: Univariate meta-regression analysis of proportion of male; Figure S15: Univariate meta-regression analysis of body mass index; Figure S16: Univariate meta-regression analysis of left ventricular ejection fraction; Figure S17: Univariate meta-regression analysis of left atrial diameter; Figure S18: Univariate meta-regression analysis of Hypertension proportion; Figure S19: Univariate meta-regression analysis of Diabetes proportion; Figure S20: Univariate meta-regression analysis of Heart failure proportion; Figure S21: Univariate meta-regression analysis of CAD proportion; Figure S22: Univariate meta-regression analysis of History of Antiarrhythmic use; Figure S23: Univariate meta-regression analysis of History of beta-blocker use; Figure S24: The Funnel Plot with Eggers test depicting the publication bias of AF recurrence by the first follow-up within 12 to 24 months post-ablation; Figure S25: The Funnel Plot with Eggers test depicting the publication bias of AF recurrences by the 6-month follow-up post-ablation; Figure S26: The Funnel Plot with Eggers test depicting the publication bias of AF recurrences by the 12-month follow-up post-ablation; Figure S27: The Funnel Plot with Eggers test depicting the publication bias of AF recurrences by the 18-month follow-up post-ablation; Figure S28: The Funnel Plot with Eggers test depicting the publication bias of AF recurrences by the 24-month follow-up post-ablation; Figure S29: The Funnel Plot with Eggers test depicting the publication bias of AF recurrences by the 36 to 42-month follow-up post-ablation; Figure S30: The Funnel Plot with Eggers test depicting the publication bias of multivariate risk of AF recurrence; Figure S31: The Funnel Plot depicting the publication bias of left atrial diameter (LAD) improvement in subjects with LAD ≥ 45 mm; Figure S32: The Funnel Plot with Eggers test depicting the publication bias of left atrial diameter (LAD) improvement in subjects with LAD < 45 mm.

## Abbreviations

SGLT2i	Sodium glucose co-transporter 2 inhibitors
HF	Heart failure
AF	Atrial fibrillation
RCTs	Randomized controlled trials
NIH	National Institutes of Health
ROB	Risk of bias
OR	Odds ratio
CI	Confidence interval
LAD	Left atrial diameter
CA	Catheter ablation
eGFR	Estimated glomerular filtration rate
LVEF	Left ventricular ejection fraction
HTN	Hypertension
T2DM	Type 2 diabetes mellitus
COPD	Chronic obstructive pulmonary disease
CVA	Cerebrovascular accident
CAD	Coronary artery disease
HLD	Hyperlipidemia
AAD	Anti-arrhythmic drugs
GLP1	Glucagon-like peptide-1
DPP4i	Dipeptidyl peptidase-4 inhibitors
PVI	Pulmonary vein isolation
CTI	Cavo-tricuspid isthmus
SVCI	Superior vena cava isolation
RFCA	Radiofrequency catheter ablation
TGF	Transforming growth factor
NHE	Sodium-hydrogen exchanger
NCX	Sodium-calcium exchanger
SERCA	Sarco/Endoplasmic Reticulum Calcium ATPase
AMPK	Adenosine monophosphate-activated protein kinase
SIRT1	Sirtuin-1
PGC-1α	Peroxisome proliferator-activated receptor-gamma coactivator 1α
IL	Interleukin
TNF	Tumor necrosis factor
CHA2DS2 VASc	Congestive heart failure, Hypertension, Age (≥75, doubled), Diabetes, Stroke/TIA (doubled), Vascular disease, Age (65–74), Sex category (Female)
