# Supplementary material for: Effects of Sodium Glucose Co-Transporter 2 Inhibitors on Atrial Fibrillation Recurrences After Catheter Ablation in Atrial Fibrillation Patients: A Systematic Review and Meta-Analysis
Source: J Clin Med. 2025 Nov 11;14(22):8001. doi: 10.3390/jcm14228001 (PMC12653339; doi:10.3390/jcm14228001)
Supplement: Supplementary file 1 [file jcm-14-08001-s001.zip › jcm-3931441-supplementary.pdf]

Study or Subgroup	SGLT2i Events	SGLT2i Total	Non-SGLT2i Events	Non-SGLT2i Total	Weight	Odds ratio M-H, Random, 95% CI
✓ Abu-Qaoud et al.	619	2225	802	2225	53.4%	0.68 [0.60 , 0.78]
✓ Hakgor et al.	41	211	144	403	18.6%	0.43 [0.29 , 0.65]
✓ Harada et al.	7	51	12	51	3.6%	0.52 [0.19 , 1.44]
✓ Kishima et al.	9	38	15	32	3.7%	0.35 [0.13 , 0.98]
✓ Liu et al.	42	45	76	77	0.8%	0.18 [0.02 , 1.83]
✓ Luo et al.	78	79	243	247	0.8%	1.28 [0.14 , 11.66]
✓ Noh et al.	29	73	103	199	11.3%	0.61 [0.36 , 1.06]
✓ Qi et al.	18	91	31	91	7.9%	0.48 [0.24 , 0.94]
✗ Zhao et al.	330	368	323	368	0.0%	1.21 [0.77 , 1.91]
**Total (HKSJ[a])**		**2813**		**3325**	**100.0%**	**0.58 [0.47 , 0.72]**
Total events:	843		1426			

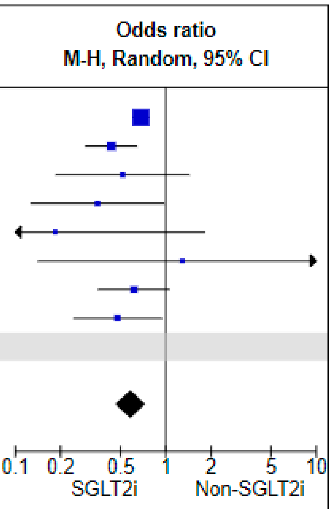

Test for overall effect: T = 5.96, df = 7 (P = 0.0006)
Test for subgroup differences: Not applicable
Heterogeneity: Tau² (DL[b]) = 0.02; Chi² = 8.49, df = 7 (P = 0.29); I² = 18%

Footnotes
[a]CI calculated by Hartung-Knapp-Sidik-Jonkman method.
[b]Tau² calculated by DerSimonian and Laird method.

Figure S1: After sensitivity analysis, AF recurrence by the final follow-up from ablation

Study or Subgroup	SGLT2i Events	SGLT2i Total	Non-SGLT2i Events	Non-SGLT2i Total	Weight	Odds ratio M-H, Random, 95% CI
✗ Abu-Qaoud et al.	619	2225	802	2225	0.0%	0.68 [0.60 , 0.78]
✓ Hakgor et al.	24	211	86	403	17.7%	0.47 [0.29 , 0.77]
✓ Harada et al.	7	51	12	51	4.0%	0.52 [0.19 , 1.44]
✓ Kishima et al.	9	38	15	32	4.0%	0.35 [0.13 , 0.98]
✓ Liu et al.	14	45	25	77	6.7%	0.94 [0.43 , 2.07]
✓ Luo et al.	18	79	81	247	12.1%	0.60 [0.34 , 1.09]
✓ Noh et al.	13	73	73	199	9.5%	0.37 [0.19 , 0.73]
✓ Qi et al.	18	91	31	91	9.2%	0.48 [0.24 , 0.94]
✓ Zhao et al.	72	368	120	368	36.8%	0.50 [0.36 , 0.70]
**Total (HKSJ[a])**		**956**		**1468**	**100.0%**	**0.51 [0.42 , 0.61]**
Total events:	175		443			

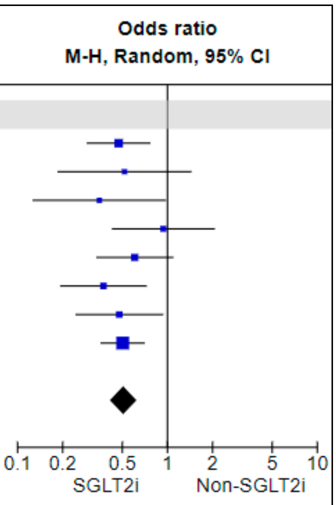

Test for overall effect: T = 8.52, df = 7 (P < 0.0001)
Test for subgroup differences: Not applicable
Heterogeneity: Tau² (DL[b]) = 0.00; Chi² = 4.09, df = 7 (P = 0.77); I² = 0%

Footnotes
[a]CI calculated by Hartung-Knapp-Sidik-Jonkman method.
[b]Tau² calculated by DerSimonian and Laird method.

Figure S2: After sensitivity analysis, AF recurrence by the first follow-up within 12 to 24 months post-ablation

Study or Subgroup	SGLT2i Events	SGLT2i Total	Non-SGLT2i Events	Non-SGLT2i Total	Weight	Odds ratio M-H, Random, 95% CI
✓ Hakgor et al.	12	211	35	403	20.4%	0.63 [0.32 , 1.25]
✓ Harada et al.	2	51	7	51	3.6%	0.26 [0.05 , 1.30]
✓ Kishima et al.	7	38	12	32	7.9%	0.38 [0.13 , 1.12]
✓ Luo et al.	10	79	46	247	17.3%	0.63 [0.30 , 1.32]
✕ Noh et al.	5	73	51	199	0.0%	0.21 [0.08 , 0.56]
✓ Zhao et al.	42	368	56	368	50.9%	0.72 [0.47 , 1.10]
**Total (HKSJ[a])**		**747**		**1101**	**100.0%**	**0.63 [0.45 , 0.88]**
Total events:	73		156			

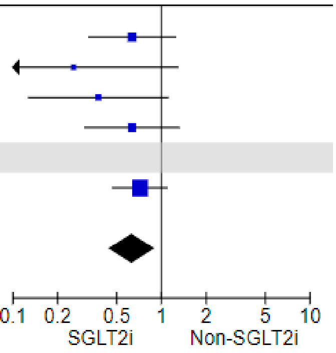

Test for overall effect: T = 3.86, df = 4 (P = 0.02)
Test for subgroup differences: Not applicable
Heterogeneity: Tau² (DL[b]) = 0.00; Chi² = 2.39, df = 4 (P = 0.66); I² = 0%

Footnotes
[a]CI calculated by Hartung-Knapp-Sidik-Jonkman method.
[b]Tau² calculated by DerSimonian and Laird method.

Figure S3: After sensitivity analysis, AF recurrences by the 6-month follow-up

Study or Subgroup	SGLT2i Events	SGLT2i Total	Non-SGLT2i Events	Non-SGLT2i Total	Weight	Odds ratio M-H, Random, 95% CI
✕ Abu-Qaoud et al.	619	2225	802	2225	0.0%	0.68 [0.60 , 0.78]
✓ Hakgor et al.	24	211	86	403	18.9%	0.47 [0.29 , 0.77]
✓ Harada et al.	7	51	12	51	4.3%	0.52 [0.19 , 1.44]
✓ Kishima et al.	9	38	15	32	4.3%	0.35 [0.13 , 0.98]
✓ Luo et al.	18	79	81	247	13.0%	0.60 [0.34 , 1.09]
✓ Noh et al.	13	73	73	199	10.2%	0.37 [0.19 , 0.73]
✓ Qi et al.	18	91	31	91	9.9%	0.48 [0.24 , 0.94]
✓ Zhao et al.	72	368	120	368	39.5%	0.50 [0.36 , 0.70]
**Total (HKSJ[a])**		**911**		**1391**	**100.0%**	**0.48 [0.42 , 0.55]**
Total events:	161		418			

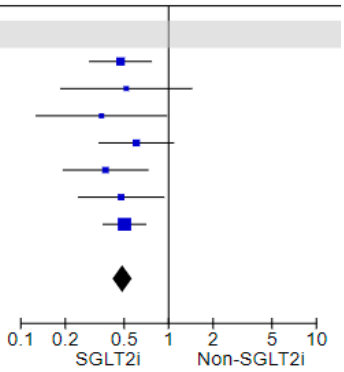

Test for overall effect: T = 13.07, df = 6 (P < 0.0001)
Test for subgroup differences: Not applicable
Heterogeneity: Tau² (DL[b]) = 0.00; Chi² = 1.58, df = 6 (P = 0.95); I² = 0%

Footnotes
[a]CI calculated by Hartung-Knapp-Sidik-Jonkman method.
[b]Tau² calculated by DerSimonian and Laird method.

Figure S4: After sensitivity analysis, AF recurrences by the 12-month follow-up

Study or Subgroup	SGLT2i Events	SGLT2i Total	Non-SGLT2i Events	Non-SGLT2i Total	Weight	Odds ratio M-H, Random, 95% CI
✓ Hakgor et al.	35	211	120	403	27.4%	0.47 [0.31 , 0.71]
✕ Luo et al.	44	79	150	247	0.0%	0.81 [0.49 , 1.36]
✓ Noh et al.	29	73	103	199	16.3%	0.61 [0.36 , 1.06]
✓ Zhao et al.	152	368	217	368	56.3%	0.49 [0.37 , 0.66]
**Total (HKSJ[a])**		**652**		**970**	**100.0%**	**0.50 [0.38 , 0.66]**
Total events:	216		440			

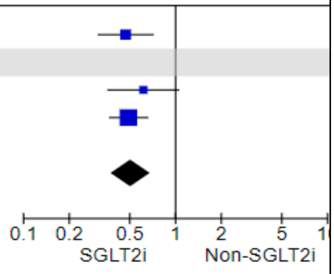

Test for overall effect: T = 10.71, df = 2 (P = 0.009)
Test for subgroup differences: Not applicable
Heterogeneity: Tau² (DL[b]) = 0.00; Chi² = 0.65, df = 2 (P = 0.72); I² = 0%

Footnotes
[a]CI calculated by Hartung-Knapp-Sidik-Jonkman method.
[b]Tau² calculated by DerSimonian and Laird method.

Figure S5: After sensitivity analysis, AF recurrences by the 18-month follow-up

Study or Subgroup	SGLT2i Events	SGLT2i Total	Non-SGLT2i Events	Non-SGLT2i Total	Weight	Odds ratio M-H, Random, 95% CI
✓ Hakgor et al	41	211	144	403	37.0%	0.43 [0.29 , 0.65]
✓ Liu et al	14	45	25	77	14.2%	0.94 [0.43 , 2.07]
✕ luo et al	64	79	197	247	0.0%	1.08 [0.57 , 2.06]
✓ Zhao et al	157	368	233	368	48.7%	0.43 [0.32 , 0.58]
**Total (HKSJ[a])**		**624**		**848**	**100.0%**	**0.48 [0.21 , 1.10]**
Total events:	212		402			

Test for overall effect: T = 3.80, df = 2 (P = 0.06)
Test for subgroup differences: Not applicable
Heterogeneity: Tau^2 (DL[b]) = 0.03; Chi^2 = 3.40, df = 2 (P = 0.18); I^2 = 41%

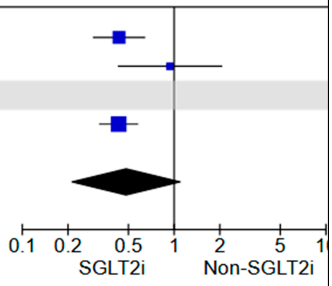

Footnotes
[a]CI calculated by Hartung-Knapp-Sidik-Jonkman method.
[b]Tau^2 calculated by DerSimonian and Laird method.

Figure S6: After sensitivity analysis, AF recurrences by the 24-month follow-up

Study or Subgroup	log[HR]	SE	Weight	Hazard ratio IV, Random, 95% CI
✓ Hakgor et al.	-0.830113	0.215258	20.9%	0.44 [0.29 , 0.66]
✓ Liu et al.	-1.714798	0.761023	1.7%	0.18 [0.04 , 0.80]
✓ Luo et al.	-0.492658	0.243654	16.3%	0.61 [0.38 , 0.99]
✕ Noh et al.	-1.89712	0.387718	0.0%	0.15 [0.07 , 0.32]
✓ Qi et al.	-0.430783	0.277208	12.6%	0.65 [0.38 , 1.12]
✓ Zhao et al.	-0.597837	0.14102	48.6%	0.55 [0.42 , 0.73]
**Total (HKSJ[a])**			**100.0%**	**0.53 [0.41 , 0.70]**

Test for overall effect: T = 6.56, df = 4 (P = 0.003)
Test for subgroup differences: Not applicable
Heterogeneity: Tau^2 (DL[b]) = 0.00; Chi^2 = 3.78, df = 4 (P = 0.44); I^2 = 0%

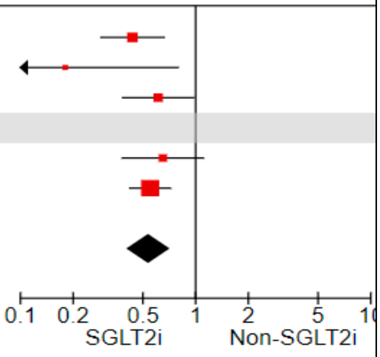

Footnotes
[a]CI calculated by Hartung-Knapp-Sidik-Jonkman method.
[b]Tau^2 calculated by DerSimonian and Laird method.

Figure S7: After sensitivity analysis, 1st Multivariate risk of AF recurrence.

Study or Subgroup	log[HR]	SE	Weight	Hazard ratio IV, Random, 95% CI
Hakgor et al	-1.155183	0.195775	21.1%	0.31 [0.21 , 0.46]
Liu et al	-1.714798	0.761023	5.6%	0.18 [0.04 , 0.80]
luo et al	-0.492658	0.243654	19.0%	0.61 [0.38 , 0.99]
Noh et al	-1.89712	0.387718	13.4%	0.15 [0.07 , 0.32]
Qi et al	-0.430783	0.277208	17.6%	0.65 [0.38 , 1.12]
Zhao et al	-0.597837	0.14102	23.2%	0.55 [0.42 , 0.73]
**Total (HKSJ[a])**			**100.0%**	**0.41 [0.22 , 0.74]**

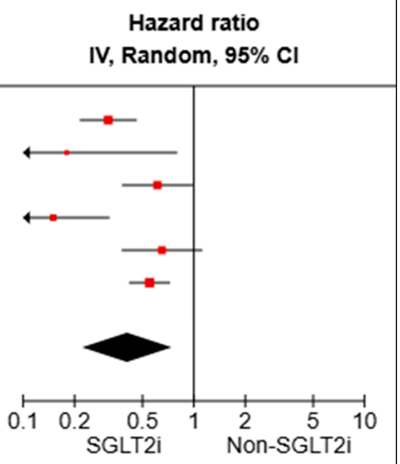

Test for overall effect: T = 3.84, df = 5 (P = 0.01)

Heterogeneity: Tau² (DL[b], 95% CI) = 0.16 [0.03 , 2.30]; Chi² = 18.13, df = 5 (P = 0.003); I² = 72%

Footnotes

[a]CI calculated by Hartung-Knapp-Sidik-Jonkman method.

[b]Tau² calculated by DerSimonian and Laird method.

Figure S8: Statistical analysis and forest plot of 2nd Multivariate risk of AF recurrence.

Study or Subgroup	log[HR]	SE	Weight	Hazard ratio IV, Random, 95% CI
Hakgor et al.	-0.830113	0.215258	20.8%	0.44 [0.29 , 0.66]
Liu et al.	-1.714798	0.761023	4.8%	0.18 [0.04 , 0.80]
luo et al.	-0.48776	0.23546	19.6%	0.61 [0.39 , 0.97]
Noh et al.	-1.89712	0.387718	12.5%	0.15 [0.07 , 0.32]
Qi et al.	-0.430783	0.277208	17.4%	0.65 [0.38 , 1.12]
Zhao et al.	-0.597837	0.14102	24.9%	0.55 [0.42 , 0.73]
**Total (HKSJ[a])**			**100.0%**	**0.44 [0.25 , 0.78]**

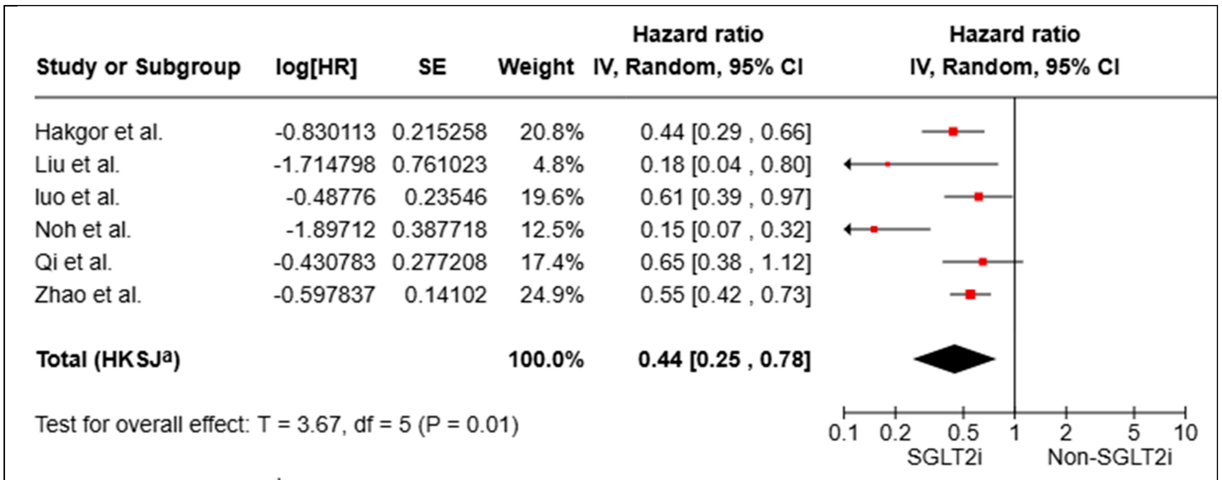

Test for overall effect: T = 3.67, df = 5 (P = 0.01)

Heterogeneity: Tau² (DL[b]) = 0.11; Chi² = 13.96, df = 5 (P = 0.02); I² = 64%

Footnotes

[a]CI calculated by Hartung-Knapp-Sidik-Jonkman method.

[b]Tau² calculated by DerSimonian and Laird method.

Figure S9: Statistical analysis and forest plot of 3nd Multivariate risk of AF recurrence.

Study or Subgroup	log[HR]	SE	Weight	Hazard ratio IV, Random, 95% CI	Hazard ratio IV, Random, 95% CI
Hakgor et al.	-1.155183	0.195775	21.0%	0.31 [0.21 , 0.46]	
Liu et al.	-1.714798	0.761023	5.6%	0.18 [0.04 , 0.80]	
luo et al.	-0.48776	0.23546	19.3%	0.61 [0.39 , 0.97]	
Noh et al.	-1.89712	0.387718	13.4%	0.15 [0.07 , 0.32]	
Qi et al.	-0.430783	0.277208	17.6%	0.65 [0.38 , 1.12]	
Zhao et al.	-0.597837	0.14102	23.1%	0.55 [0.42 , 0.73]	
**Total (HKSJ[a])**			**100.0%**	**0.41 [0.22 , 0.74]**	

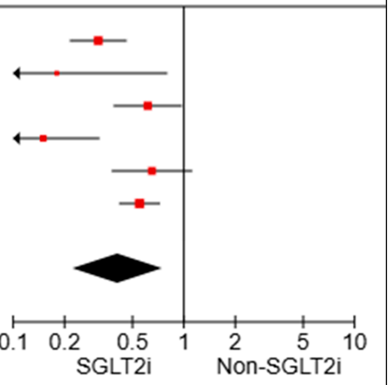

Test for overall effect: T = 3.83, df = 5 (P = 0.01)

Heterogeneity: Tau^2 (DL[b]) = 0.16; Chi^2 = 18.28, df = 5 (P = 0.003); I^2 = 73%

Footnotes

[a]CI calculated by Hartung-Knapp-Sidik-Jonkman method.

[b]Tau^2 calculated by DerSimonian and Laird method.

Figure S10: Statistical analysis and forest plot of 4th Multivariate risk of AF recurrence.

Study or Subgroup	log[HR]	SE	Weight	Hazard ratio IV, Random, 95% CI	Hazard ratio IV, Random, 95% CI
Hakgor et al.	-0.673345	0.227293	41.3%	0.51 [0.33 , 0.80]	
Zhao et al.	-0.634878	0.190619	58.7%	0.53 [0.36 , 0.77]	
**Total (HKSJ[a])**			**100.0%**	**0.52 [0.41 , 0.66]**	

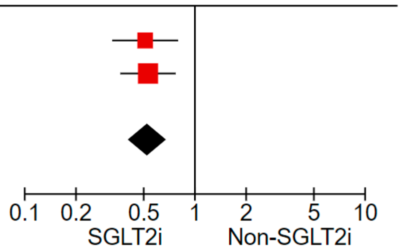

Test for overall effect: T = 34.36, df = 1 (P = 0.02)

Test for subgroup differences: Not applicable

Heterogeneity: Tau^2 (DL[b]) = 0.00; Chi^2 = 0.02, df = 1 (P = 0.90); I^2 = 0%

Footnotes

[a]CI calculated by Hartung-Knapp-Sidik-Jonkman method.

[b]Tau^2 calculated by DerSimonian and Laird method.

Figure S11: Statistical analysis and forest plot illustrates the hazard ratio of left atrial diameter (LAD) improvement in subjects with LAD >= 45 mm receiving Sodium-glucose cotransporter 2 inhibitor (SGLT2i) vs non- SGLT2i across studies. Squares represent individual study estimates, sized by study weight; horizontal lines show 95% confidence interval (CI). The diamond denotes the pooled effect estimate and its 95% CI.

Study or Subgroup	log[HR]	SE	Weight	Hazard ratio IV, Random, 95% CI
Hakgor et al.	-1.049822	0.309813	42.0%	0.35 [0.19 , 0.64]
Zhao et al.	-0.478036	0.208939	58.0%	0.62 [0.41 , 0.93]
**Total (HKSJ[a])**			**100.0%**	**0.49 [0.01 , 17.59]**

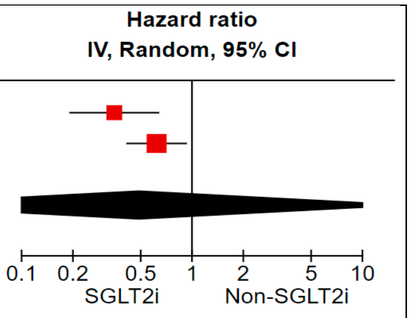

Test for overall effect: T = 2.54, df = 1 (P = 0.24)
Test for subgroup differences: Not applicable
Heterogeneity: Tau² (DL[b]) = 0.09; Chi² = 2.34, df = 1 (P = 0.13); I² = 57%

Footnotes
[a]CI calculated by Hartung-Knapp-Sidik-Jonkman method.
[b]Tau² calculated by DerSimonian and Laird method.

Figure S12: Statistical analysis and forest plot illustrates the hazard ratio of left atrial diameter (LAD) improvement in subjects with LAD < 45 mm receiving Sodium-glucose cotransporter 2 inhibitor (SGLT2i) vs non- SGLT2i across studies. Squares represent individual study estimates, sized by study weight; horizontal lines show 95% confidence interval (CI). The diamond denotes the pooled effect estimate and its 95% CI.

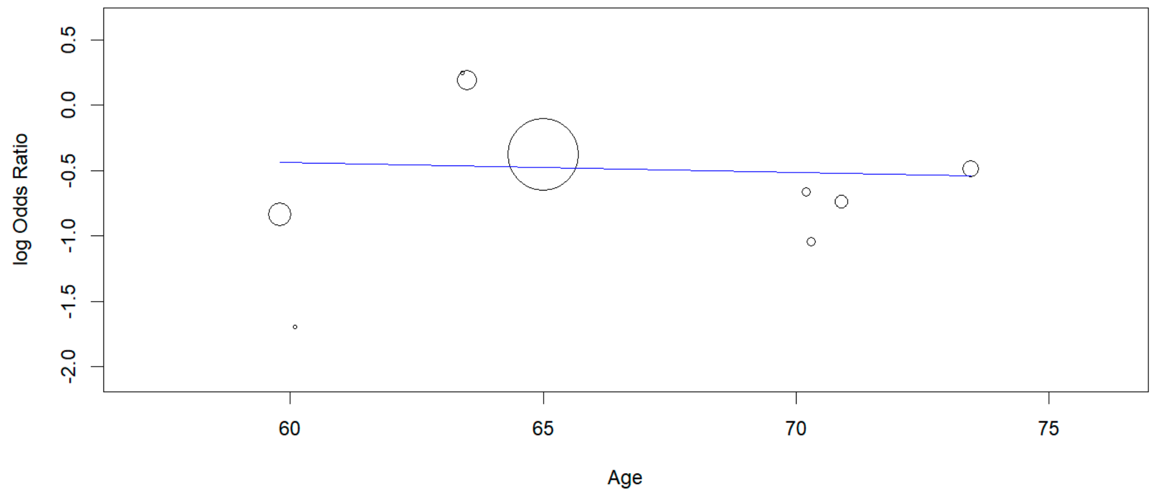

Figure S13: Univariate meta-regression analysis of Age.

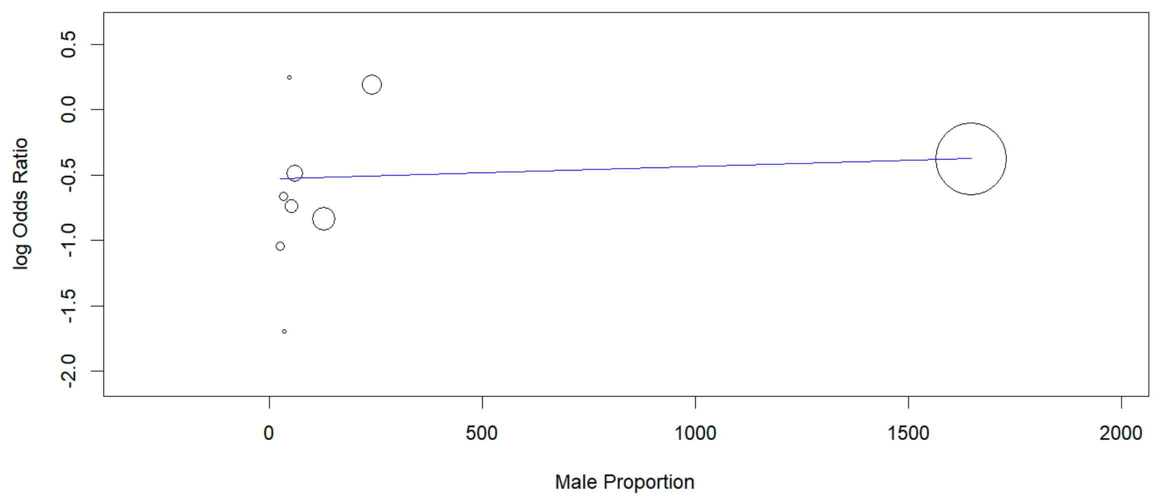

Figure S14: Univariate meta-regression analysis of proportion of male.

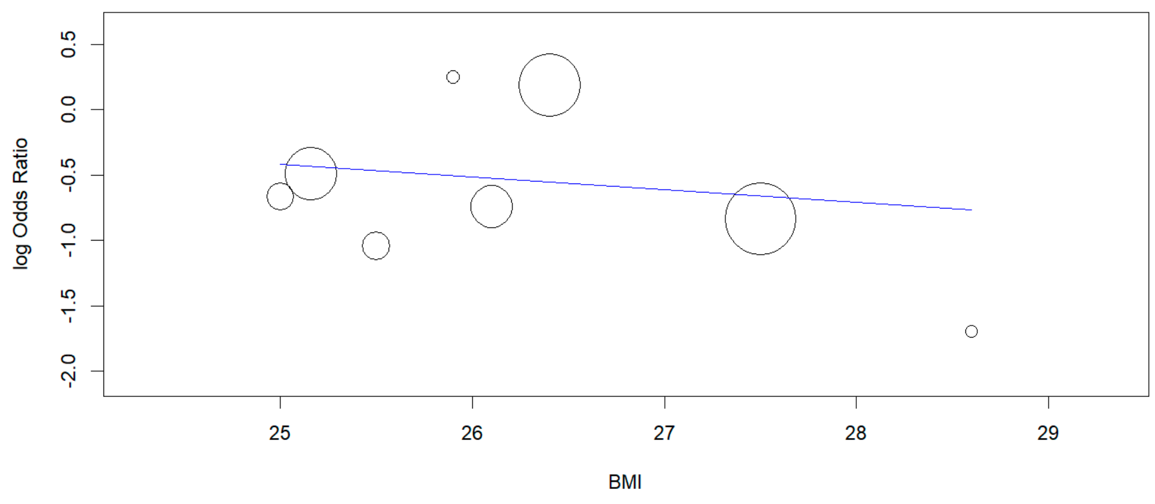

Figure S15: Univariate meta-regression analysis of body mass index.

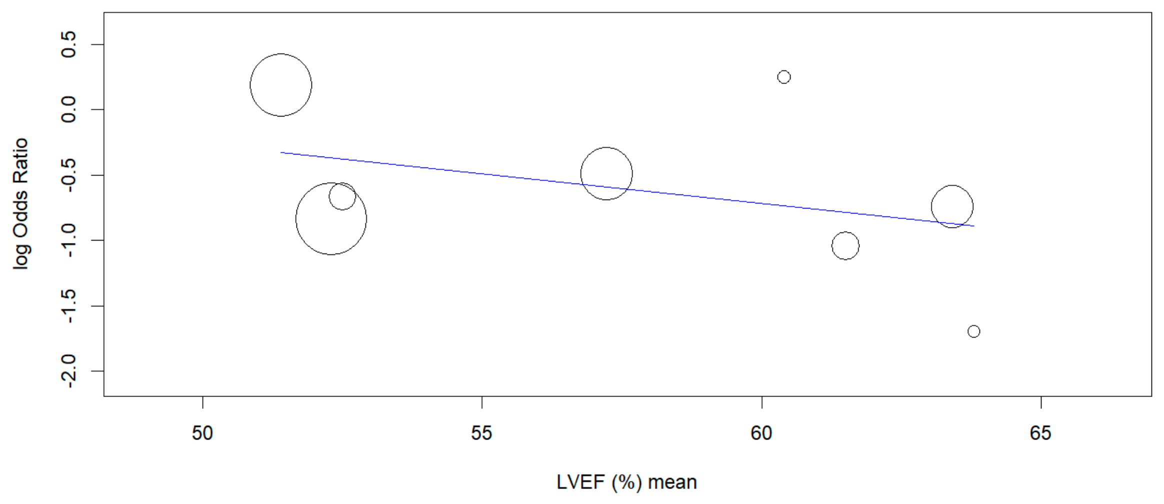

Figure S16: Univariate meta-regression analysis of left ventricular ejection fraction.

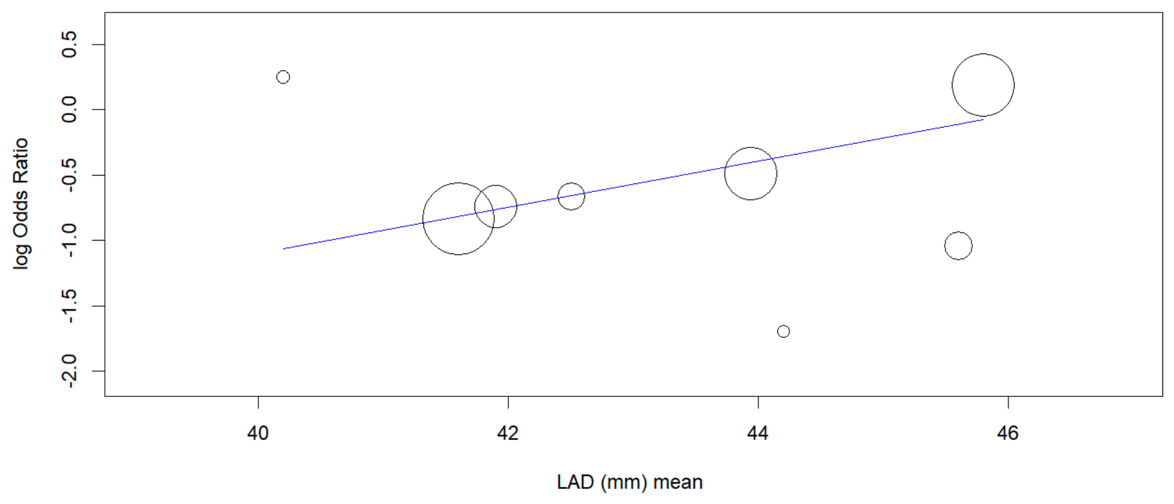

Figure S17: Univariate meta-regression analysis of left atrial diameter.

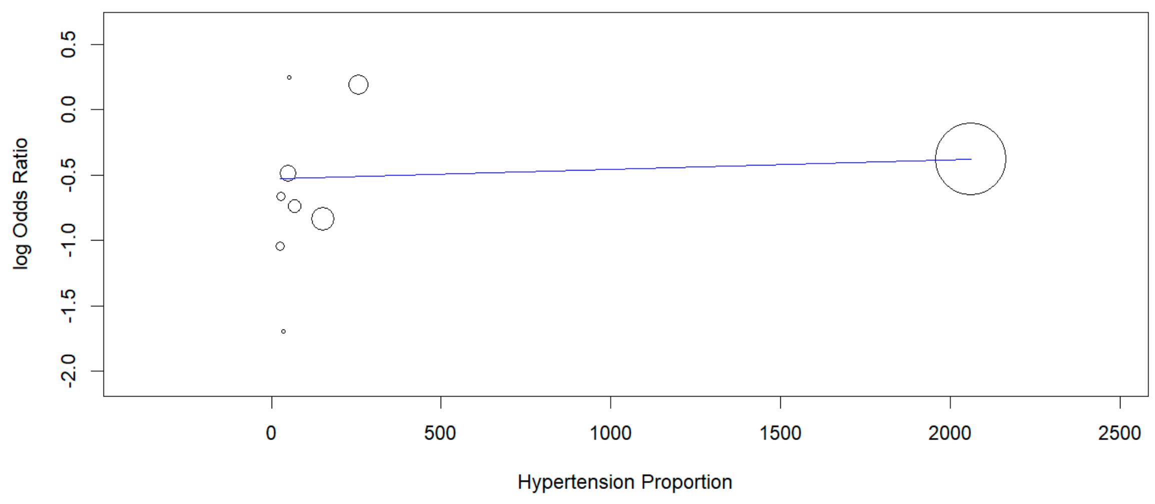

Figure S18: Univariate meta-regression analysis of Hypertension proportion.

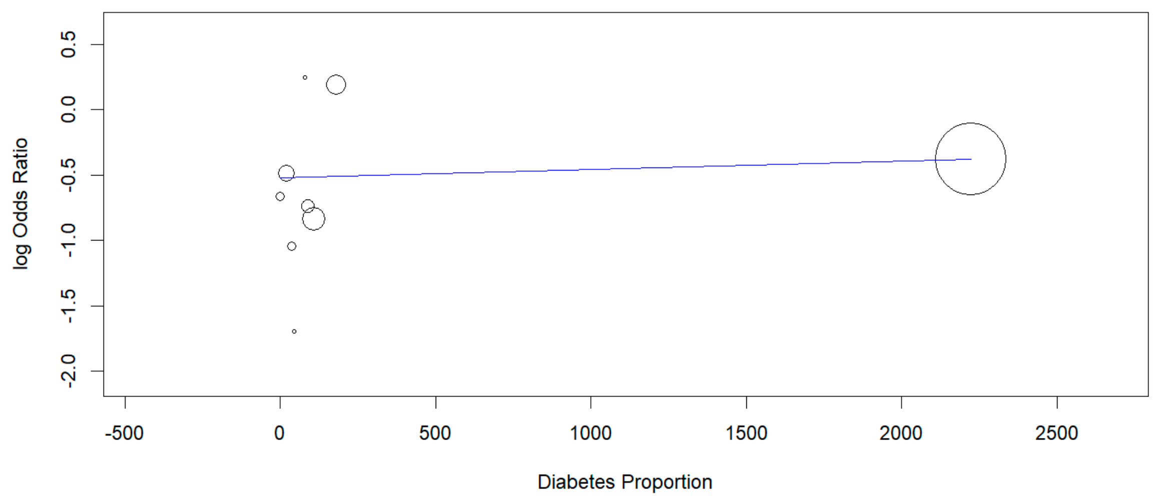

Figure S19: Univariate meta-regression analysis of Diabetes proportion.

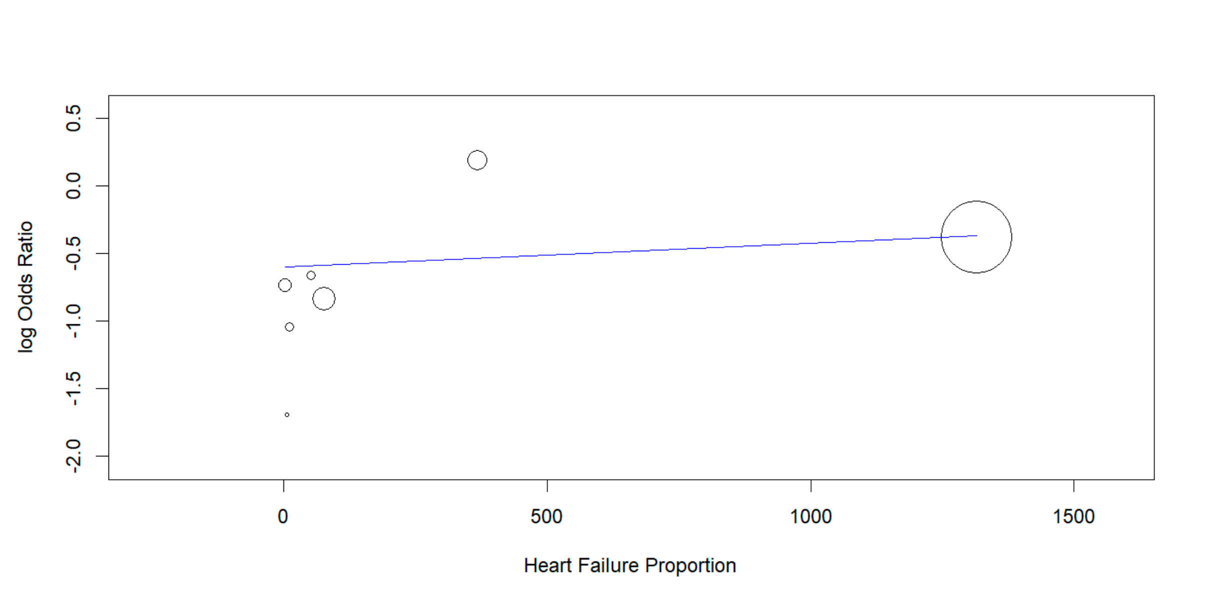

Figure S20: Univariate meta-regression analysis of Heart failure proportion.

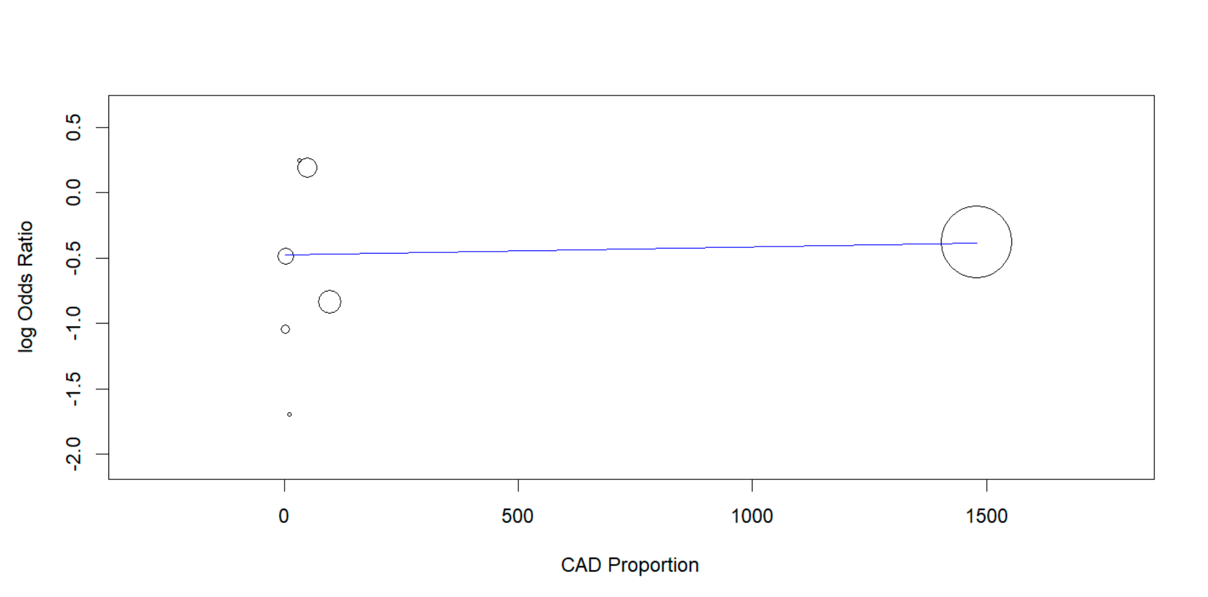

Figure S21: Univariate meta-regression analysis of CAD proportion.

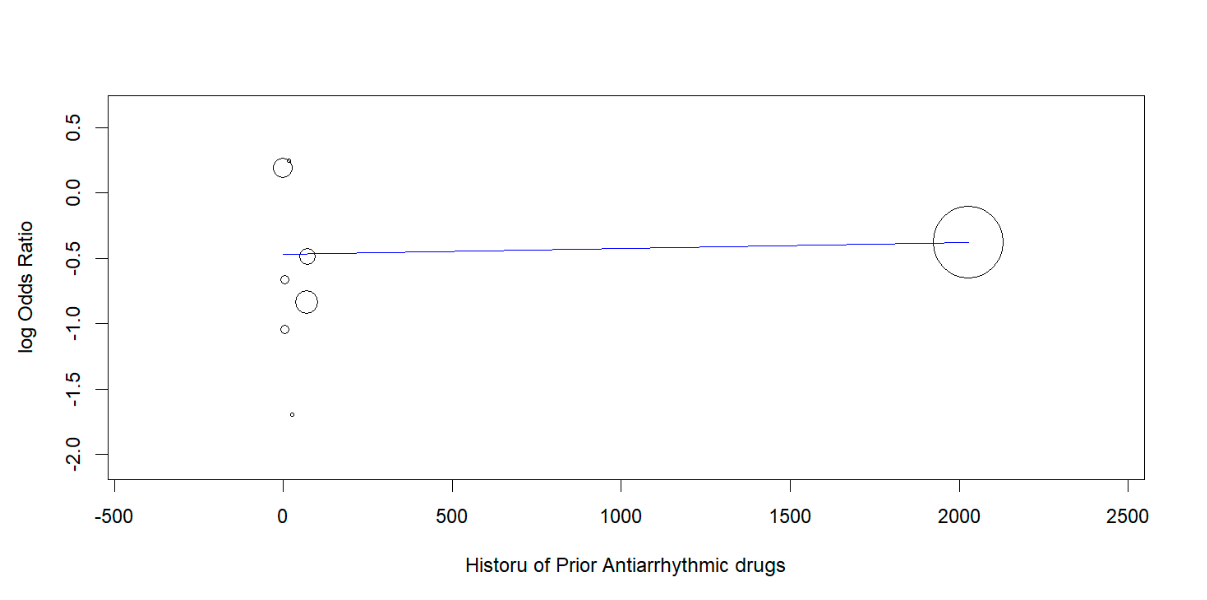

Figure S22: Univariate meta-regression analysis of History of Antiarrhythmic use.

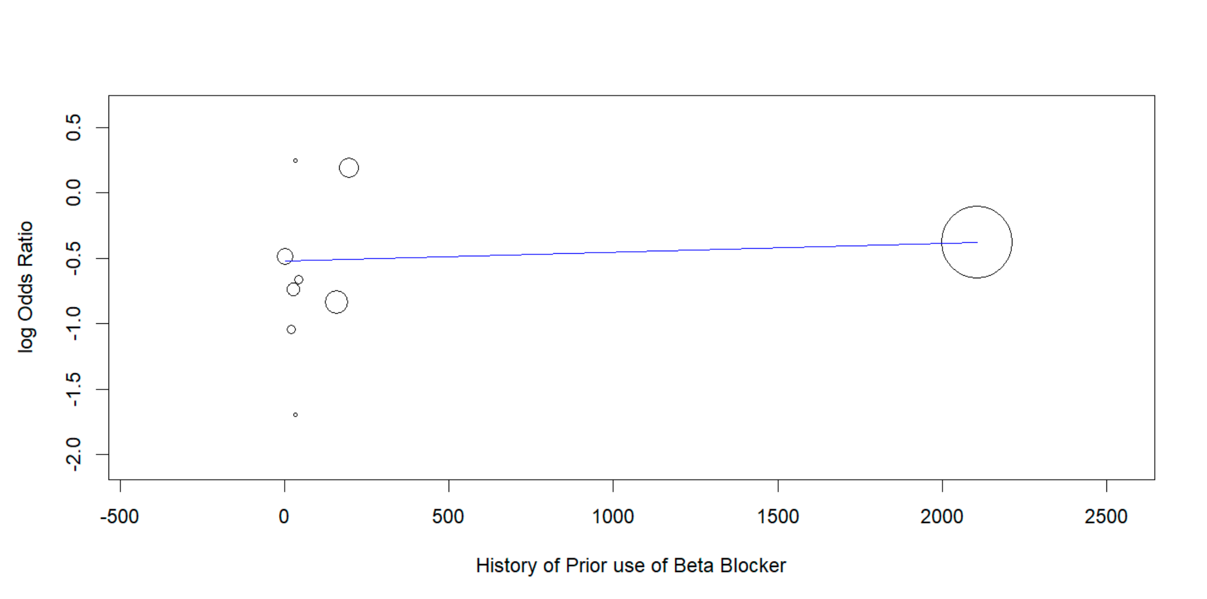

Figure S23: Univariate meta-regression analysis of History of beta-blocker use.

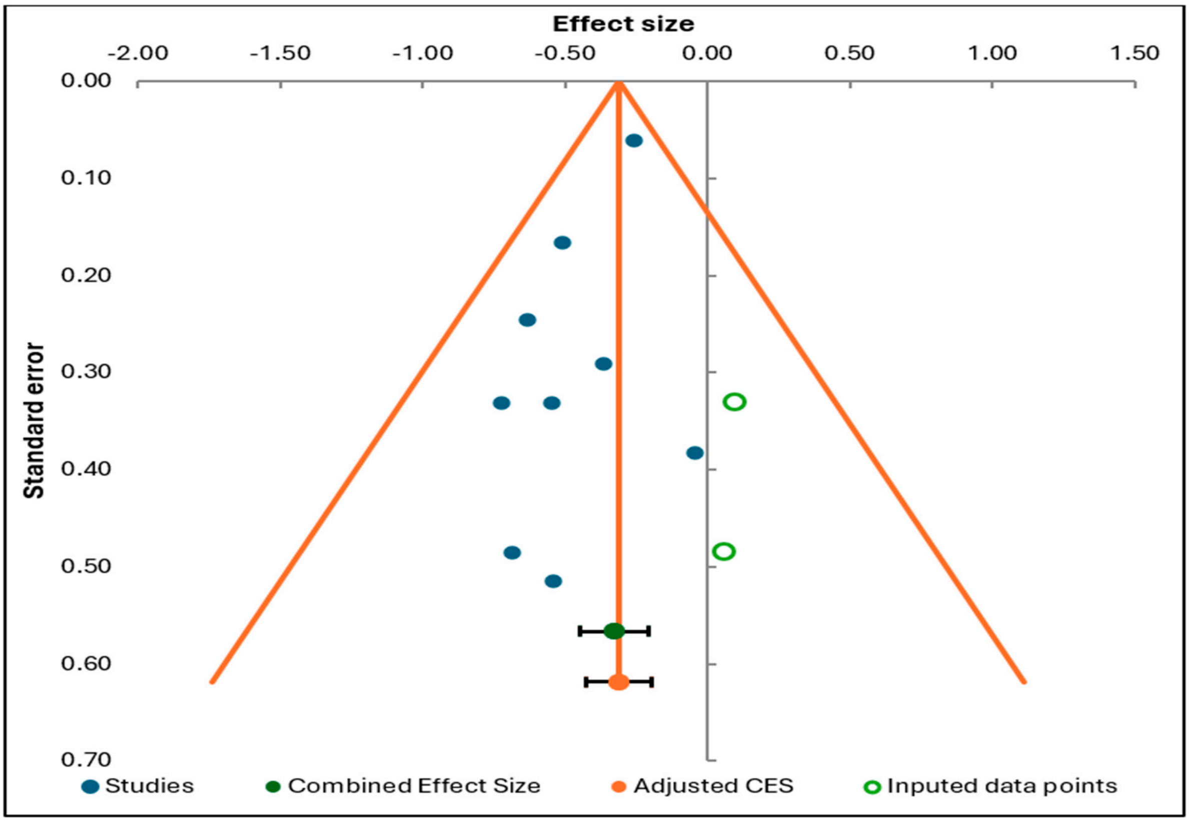

Egger Regression						
	**Estimate**	**SE**	**CI LL**	**CI UL**	**t test**	**p-value**
**Intercept**	-0.89	0.37	-1.75	-0.04	**-2.42**	**0.046***
**Slope**	-0.22	0.06	-0.36	-0.09		

Figure S24: The Funnel Plot with Eggers test depicting the publication bias of AF recurrence by the first follow-up within 12 to 24 months post-ablation.

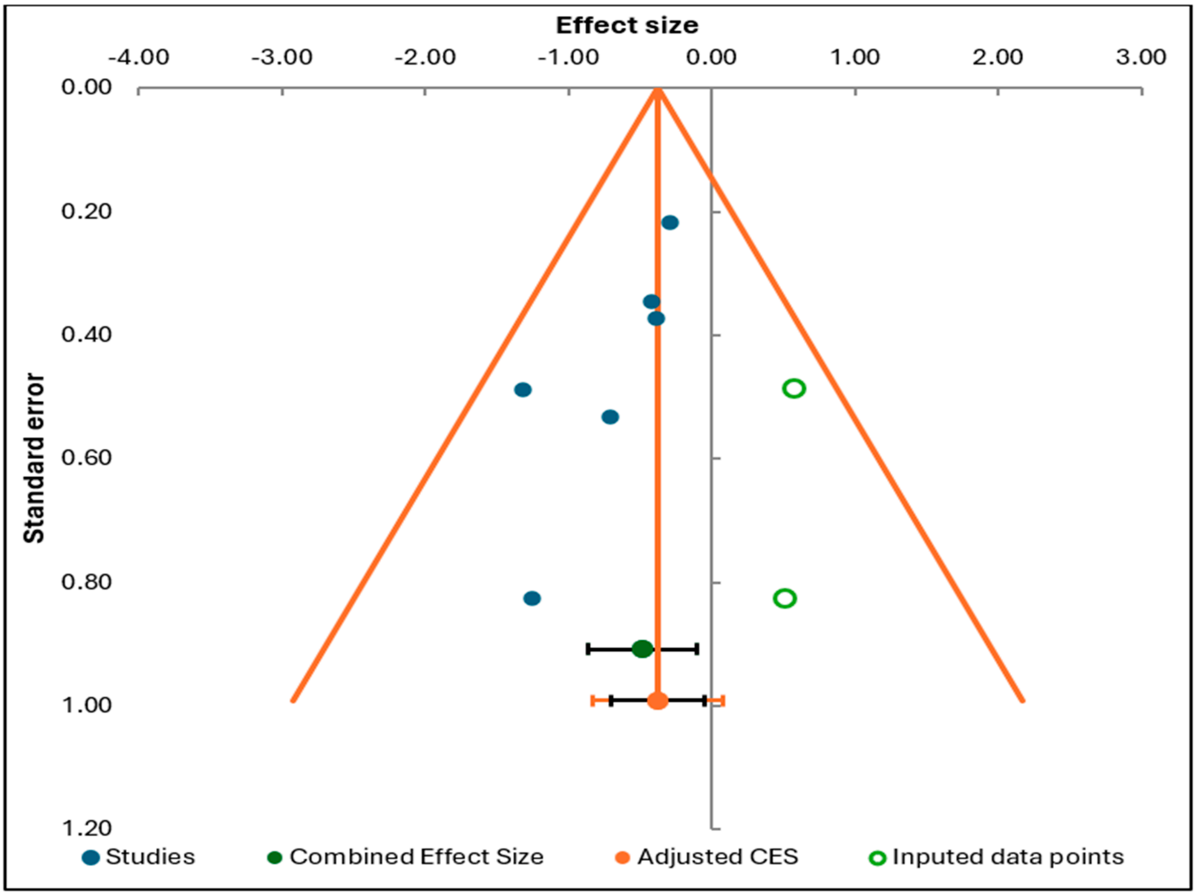

Egger Regression						
	**Estimate**	**SE**	**CI LL**	**CI UL**	**t test**	**p-value**
**Intercept**	-1.91	0.68	-3.64	-0.17	**-2.82**	**0.048***
**Slope**	0.15	0.24	-0.48	0.78		

Figure S25: The Funnel Plot with Eggers test depicting the publication bias of AF recurrences by the 6-month follow-up post-ablation

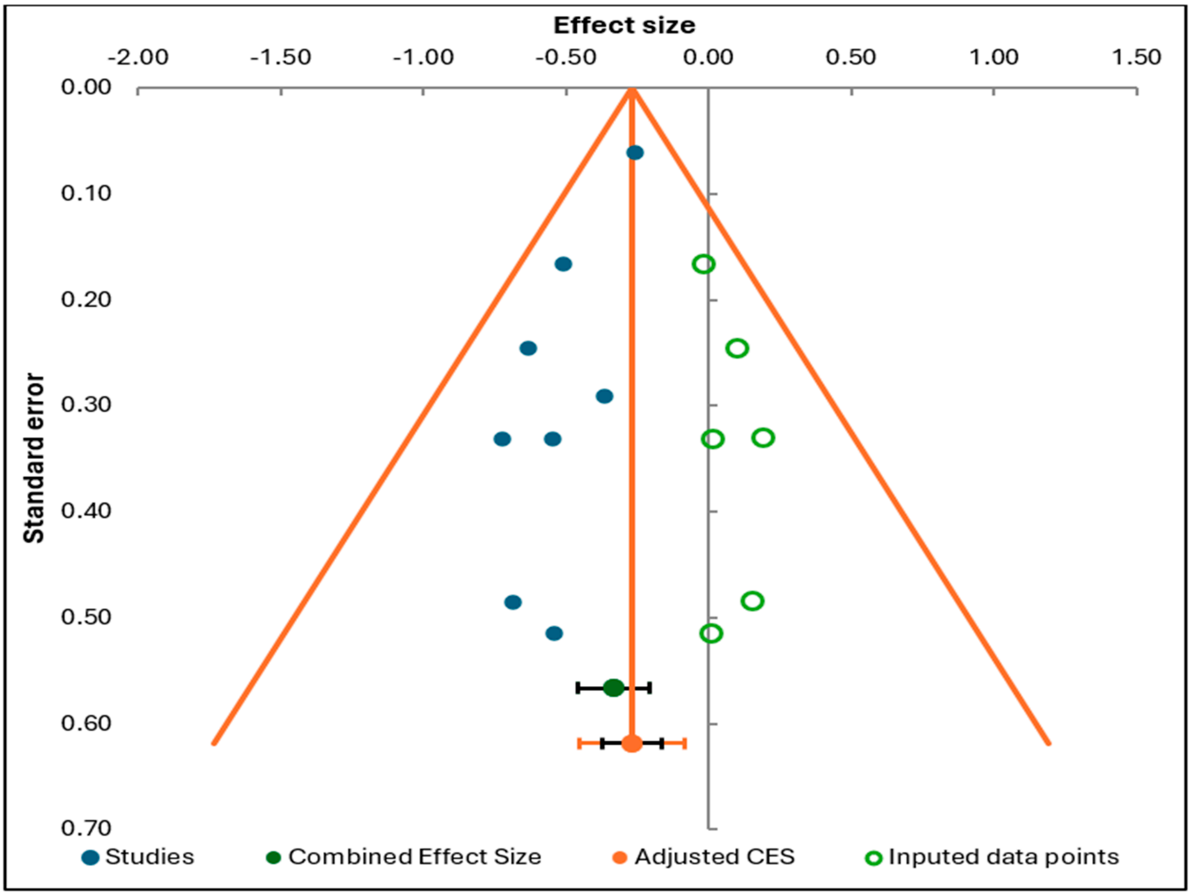

Egger Regression						
	**Estimate**	**SE**	**CI LL**	**CI UL**	**t test**	**p-value**
**Intercept**	-1.17	0.28	-1.84	-0.50	**-4.12**	**0.006***
**Slope**	-0.20	0.04	-0.30	-0.10		

Figure S26: The Funnel Plot with Eggers test depicting the publication bias of AF recurrences by the 12-month follow-up post-ablation

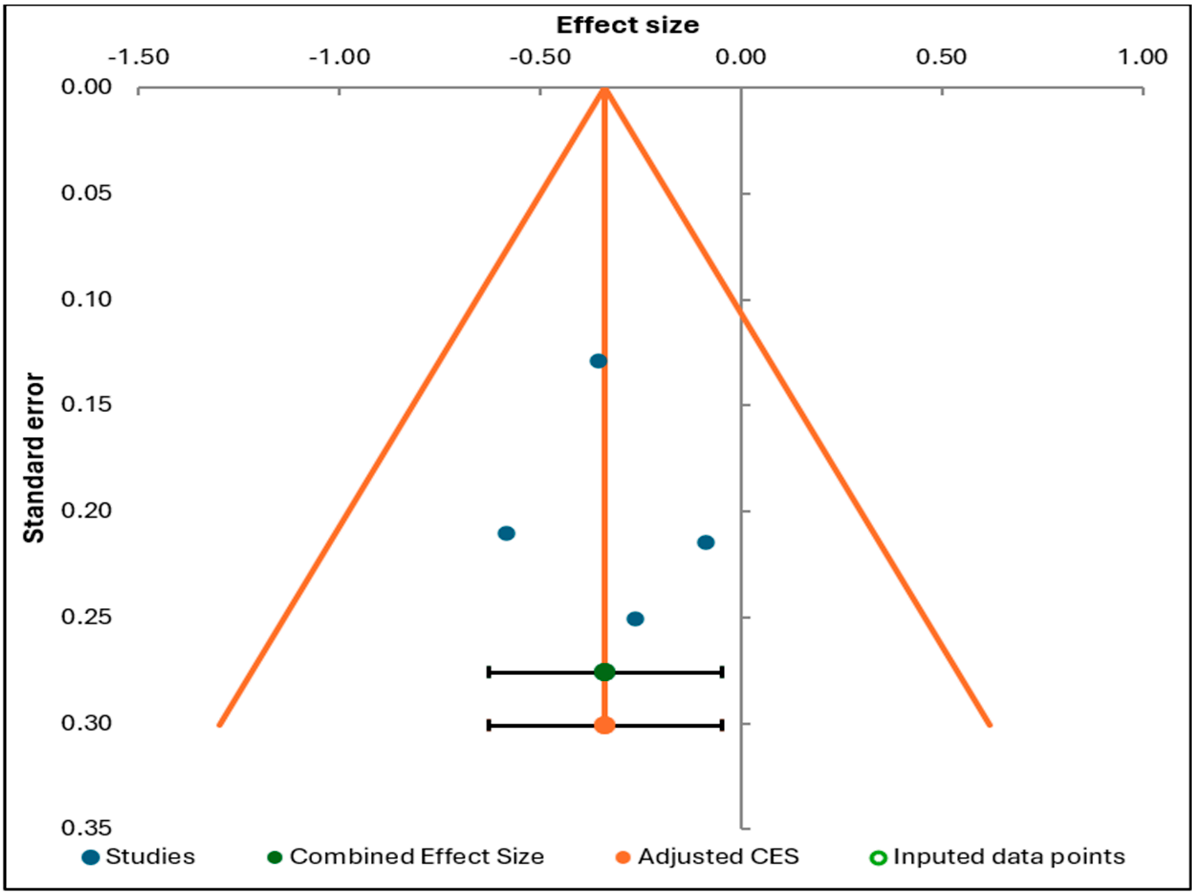

Egger Regression

	Estimate	SE	CI LL	CI UL	t test	p-value
**Intercept**	0.56	2.22	-6.50	7.63	**0.25**	**0.823**
**Slope**	-0.44	0.40	-1.73	0.85		

Figure S27: The Funnel Plot with Eggers test depicting the publication bias of AF recurrences by the 18-month follow-up post-ablation

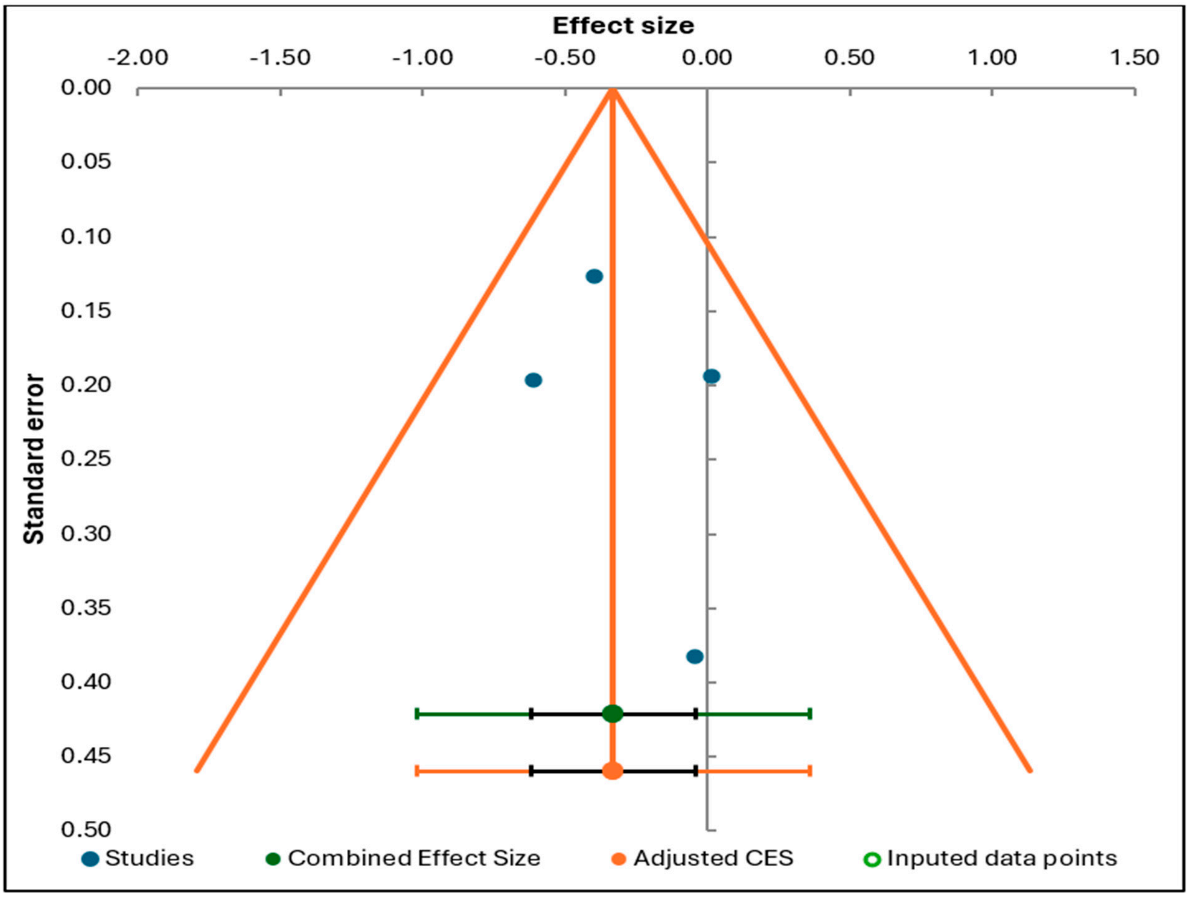

Egger Regression						
	**Estimate**	**SE**	**CI LL**	**CI UL**	**t test**	**p-value**
**Intercept**	1.35	2.39	-6.25	8.94	**0.56**	**0.629**
**Slope**	-0.56	0.43	-1.94	0.82		

Figure S28: The Funnel Plot with Eggers test depicting the publication bias of AF recurrences by the 24-month follow-up post-ablation

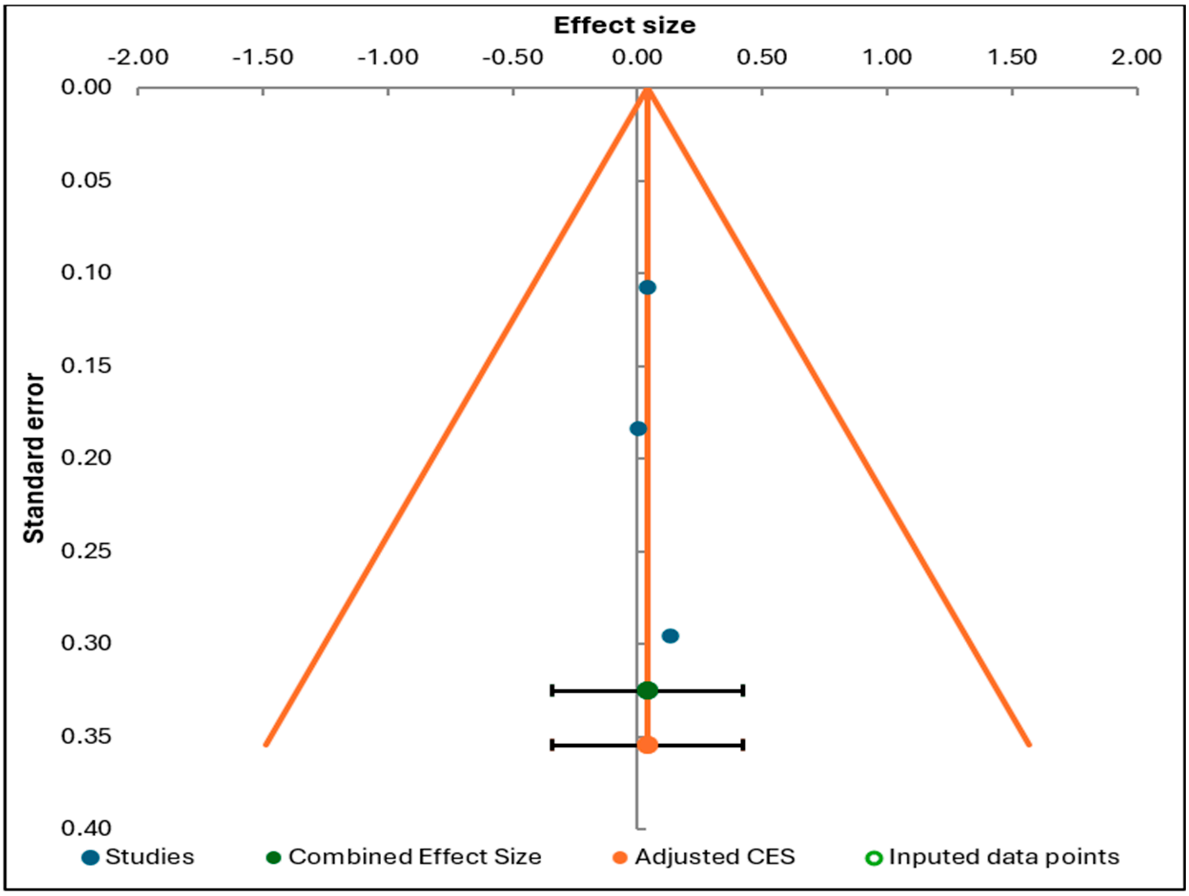

Egger Regression						
	**Estimate**	**SE**	**CI LL**	**CI UL**	**t test**	**p-value**
**Intercept**	0.27	0.50	-1.86	2.41	**0.55**	**0.678**
**Slope**	0.00	0.08	-0.33	0.33		

Figure S29: The Funnel Plot with Eggers test depicting the publication bias of AF recurrences by the 36 to 42-month follow-up post-ablation

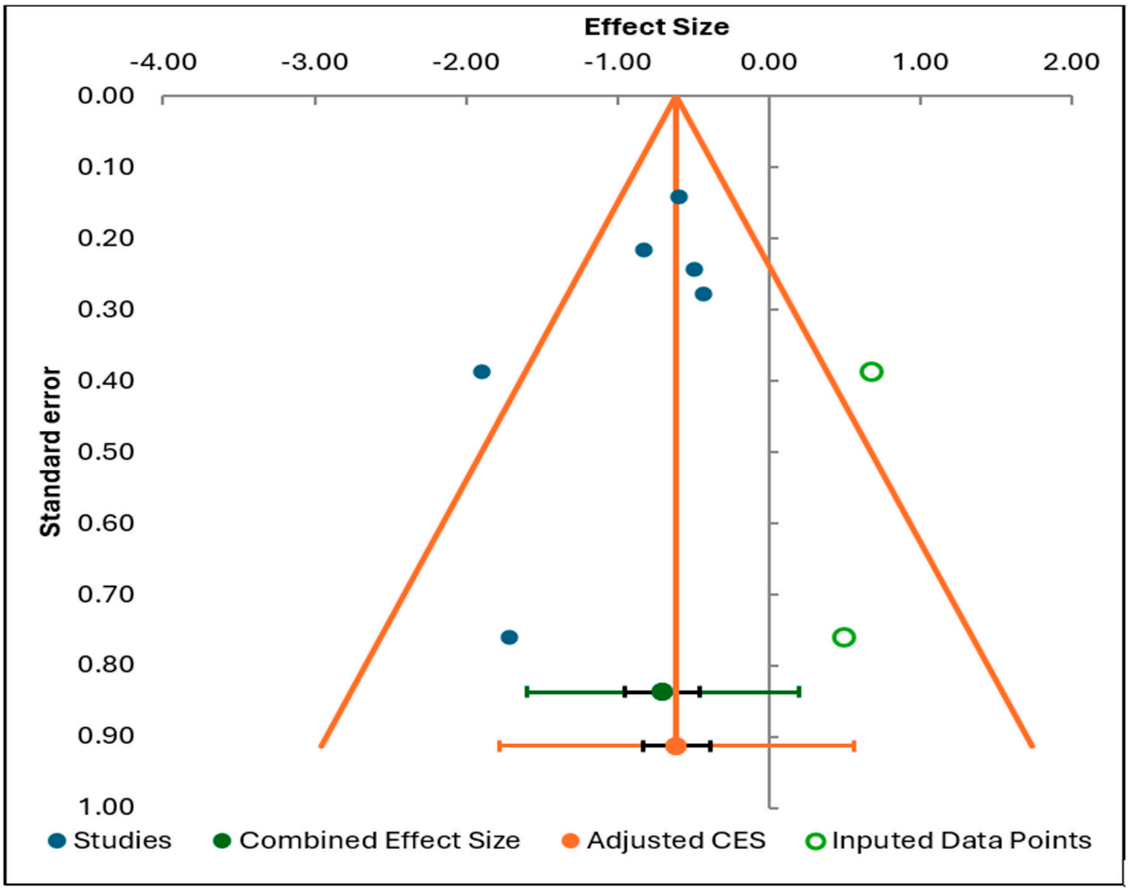

Egger Regression						
	**Estimate**	**SE**	**CI LL**	**CI UL**	**t test**	**p-value**
**Intercept**	-2.28	1.41	-5.91	1.36	**-1.61**	**0.182**
**Slope**	-0.22	0.33	-1.07	0.63		

Figure S30: The Funnel Plot with Eggers test depicting the publication bias of multivariate risk of AF recurrence

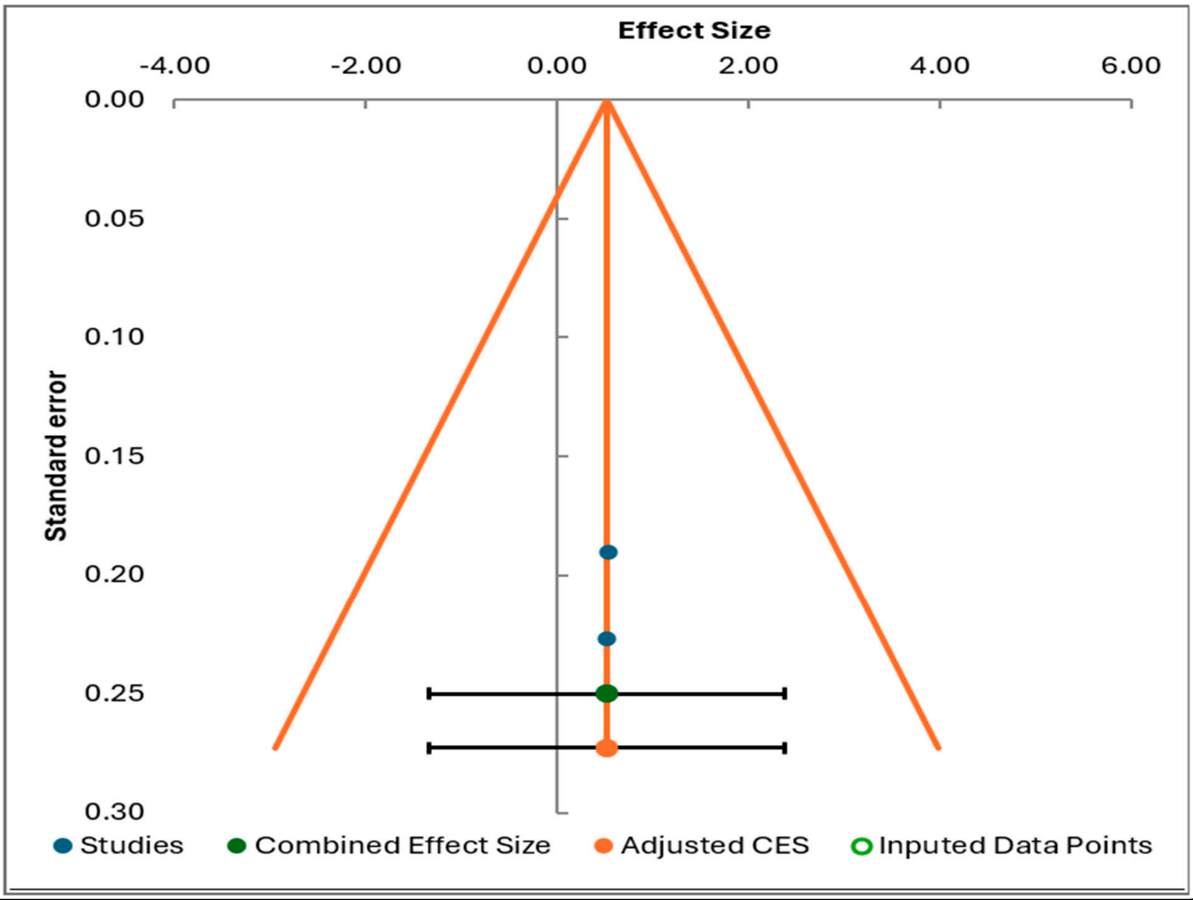

Figure S31: The Funnel Plot depicting the publication bias of left atrial diameter (LAD) improvement in subjects with LAD >= 45 mm

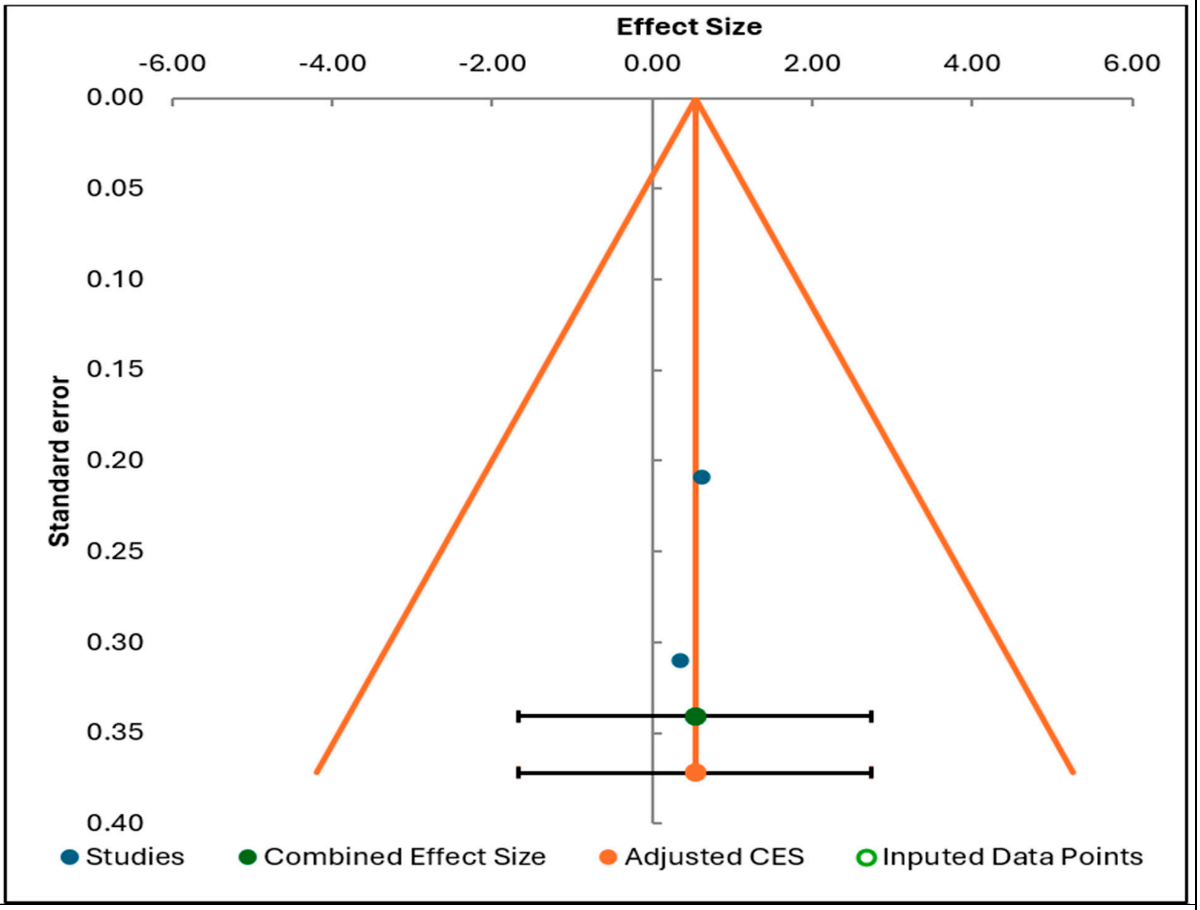

Figure S32: The Funnel Plot with Eggers test depicting the publication bias of left atrial diameter (LAD) improvement in subjects with LAD < 45 mm
